# New Chitosan Polymer Scaffold Schiff Bases as Potential Cytotoxic Activity: Synthesis, Molecular Docking, and Physiochemical Characterization

**DOI:** 10.3389/fchem.2021.796599

**Published:** 2022-01-17

**Authors:** Ponnusamy Packialakshmi, Perumal Gobinath, Daoud Ali, Saud Alarifi, Raman Gurusamy, Akbar Idhayadhulla, Radhakrishnan Surendrakumar

**Affiliations:** ^1^ Research, Department of Chemistry, Nehru Memorial College, Affiliated Bharathidasan University, Puthanampatti, India; ^2^ Department of Zoology, College of Science, King Saud University (KSU), Riyadh, Saudi Arabia; ^3^ Department of Life Sciences, Yeungnam University, Gyeongsan, South Korea

**Keywords:** schiff base, SEM, anticancer activity, MCF-7, *in-silico* molecular docking, structure activity relationship

## Abstract

In this work, we synthesize the sulfonated Schiff bases of the chitosan derivatives 2a-2j without the use of a catalyst in two moderately straightforward steps with good yield within a short reaction time. The morphology and chemical structure of chitosan derivatives were investigated using FT-IR, NMR (^1^H—^13^C), XRD, and SEM. Furthermore, our chitosan derivatives were tested for their anticancer activity against the MCF-7 cancer cell line, and doxorubicin was used as a standard. In addition, the normal cell lines of the breast cancer cell MCF-10A, and of the lung cell MRC-5 were tested. Compound 2 h, with a GI_50_ value of 0.02 µM for MCF-7, is highly active compared with the standard doxorubicin and other compounds. The synthesized compounds 2a-2j exhibit low cytotoxicity, with IC_50_ > 100 μg/ml, against normal cell lines MCF-10A, MRC-5. We also provide the results of an *in-silico* study involving the Methoxsalen protein (1Z11). Compound 2h exhibits a higher binding affinity for 1Z11 protein (−5.9 kcal/mol) and a lower binding affinity for Doxorubicin (−5.3 kcal/mol) than certain other compounds. As a result of the aforementioned findings, the use of compound 2h has an anticancer drug will be researched in the future.

## Introduction

**FIGURE 1:**
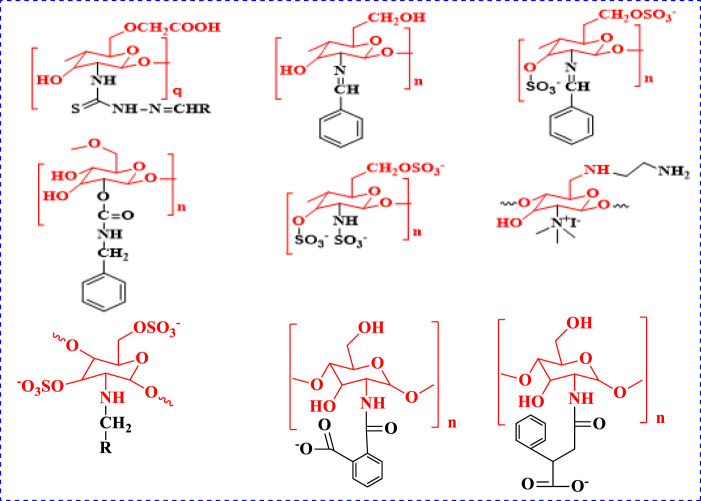
Anticancer active compounds of chitosan derivatives.

Cell proliferation becomes abnormal when it expands outside its normal limits, invades adjacent tissues, and/or feeds on other parts of the body, and can cause cancer. Cancer can begin anywhere in the body. A period of growth is the primary trigger for cancer-related death. The World Health Organization (WHO) reports that cancer remains the leading cause of death in most countries. Cancer is the second leading cause of death worldwide, with new cancer diagnoses expected to reach 18.1 million in 2018 and with deaths due to cancer expected to exceed 9.6 million. Both men and women can be affected by cancer. According to a UN DESA assessment from 2014, the world’s population, currently at 7.3 billion people, will grow to 8.5 billion by 2030, to 9.7 billion by 2050, and to 11.2 billion by 2,100. The estimate is that 1.5 billion cancer cases will bediagnosed, and that 1.2 billion fatalities will occur (Bray and Møller, 2006). In addition, with an anticipated 8.5 billion people on the planet, global mortality is expected to hit 2.14 billion by 2030 (Ali et al., 2011; Pereira et al., 2012). Cancers of the breast and lung are major causes of death in both women and men (5,22,000 deaths in 2012). In over 140 nations, these two cancers are the most often diagnosed (Ali et al., 2015). Lung cancer and female breast cancer are the two most life-threatening malignancies, according to research. Breast cancer is one of the most frequent cancers in women and one of the most deadly (Bray et al., 2018). The activities of chitosan in killing cancer cells are known to work via the induction of apoptosis due to the activation of caspases 3, 8, and 9; the modification of the BaX- to Bcl-2 ratio; and the induction of DNA damage (Adhikari and Yadav, 2018), with the features of chitosan protonation present in acidic environments. Chitosan and its derivatives have been shown to selectively permeate cancer cell membranes and exhibit anticancer activity via cellular enzymatic, antiangiogenic, immune-enhancing, antioxidant defense mechanism, and apoptotic mechanisms. This activity can be reduced in oral administration due to protonation of the amine group (Mahmood et al., 2019). Despite this, the clinical options for chitosan in the treatment of a number of human cancers remain limited (Kuppusamy and Karuppaiah, 2013). The therapeutic use of chitosan-based molecules with minimal non-cancer cell damage is vital. Chitosan-based molecules were discovered to have anticancer properties that cause minimal damage to non-cancer cells (Wimardhani et al., 2014), and its effect varies greatly depending on the molecular weight and DDA against different cancer cell lines. Many cancer cells are affected by chemotherapeutic medicines such as cisplatin, 5- fluorouracil (5-FU), docetaxel, procarbazine, methotrexate, etc. according to studies conducted *in vitro* and *in vivo* (Andreadis et al., 2003). The Schiff base approach was used to make the DCMC/SS film, which has a wide range of biological applications including wound treatment, artificial skin, and tissue engineering (Wang et al., 2019). In addition to its mucoadhesive properties, chitosan suppresses drug efflux processes, allowing for greater drug penetration into cells (Taher et al., 2019). Chitosan and its derivatives, notably chitosan-drug nanocomposites as the principal anticancer formulations, can be viable natural choices to overcome these problems due to their selective antitumor effects, nontoxicity, biocompatibility, and biodegradability. Chitosan is a nontoxic, non-allergenic, biocompatible, and biodegradable natural polyaminosaccharide deacetylated from chitin (Imran et al., 2020). Chitosan has three types of reactive groups at C-2, C-3, and C-6: the primary amine group, and the primary and secondary hydroxyl groups. Among the three reactive groups for chitosan’s biological activity, glucosamine’s main amine at C-2 is the most important group (Aranaz et al., 2010). Chitosan and chitosan compounds with higher DDA and lower MW had better antibacterial, antioxidant, and anticancer properties (Kim, 2018). Chitosan and its derivatives have good biological activity, including anticancer properties, and makes it a good carrier for the preparation of prodrugs, as an accelerator of tissue engineering, and as an excipient for drug delivery and gene delivery (Muanprasat and Chatsudthipong, 2017; Cheung et al., 2015; Zhang et al., 2002; Ahmed and Ikram, 2016; Zhang et al., 2018; Bakar et al., 2017). The biological characteristics of chitosan are influenced by its solubility in water and in other commonly used solvents (Kumirska et al., 2011). Under mild conditions, chitosan includes two types of reactive functional groups (an amino group and a hydroxyl group) that can be used to modify its characteristics and to generate other derivatives (Li et al., 2015). Chitosan has garnered a lot of interest recently because of its commercial potential in the medical, chemical, and food industries. Chitosan is frequently employed in biomedical applications due to its ability to obtain an extremely adaptable combination of chemical elements and properties as well as due to its ease of extraction, chemical modifications, and low cost (Chen and Gong, 2016). Chitosan and chitosan derivatives have good anticancer effects and decrease the unwanted effects of some medicine due to its controlled administration of doses for cancer treatment and its slow release of the free drug from conjugates (Perni and Prokopovich, 2017). Following that, efforts are required to identify newer, more effective, and less toxic chemotherapeutic drugs for the treatment of cancers (Srivastava et al., 2019). The anticancer active compounds of chitosan analogs are shown in Figure 1 (Jiang et al., 2011; Adhikari and Yadav, 2018; Li et al., 2020; Saeed et al., 2020). In recent years, CH-based tissue regeneration and drug delivery to implants as well as chitosan’s potential for use as a biomaterial in dental applications have been researched (Husain et al., 2017; Qasim et al., 2018). Chitosan is utilized in the pharmaceutical industry for making drug delivery matrixes such as blend films (CS/PAH) as well as antibacterial activities (Ali et al., 2020; Sarwar et al., 2020).

The contemporary work addresses the development of the cytotoxic activity of fresh sulfonated Schiff base of chitosan derivatives can be synthesized and characterized by FT-IR, NMR (^1^H NMR, ^13^C NMR) spectroscopy, and its physical properties were observed. *In-silico* molecular docking analysis and also evaluated the cytotoxic activities against a cancer cell lines.

## Experimental

### Materials and Methods (Chemistry)

All the substance and reagents such as chitosan (degree of deacetylation - 75%, Mw141 KDa), acetic acid, AEEA, chlorosulfonicacid, aromatic aldehydes, ethanol and DMF are purchased from Merck. The melting points of synthesized substances were evaluated in open capillary tubes, and were uncorrected. The FT-IR spectra were recorded in KBr on a Shimadzu 8201pc (4,000–1,000 cm^−1^). The ^1^H-NMR and ^13^CNMR spectra were recorded on a Bruker DRX-300 MHz. To obtain NMR spectra, the product was dissolved in DMSO-d_6_. TLC was used to assess the purity of the compounds, with silica-gel and 60F254 aluminium sheets as the adsorbent.

### Preparation of Chitosan Analogue (1, 2, 2a–2j)

Chitosan (3 g, deacetylated) was dissolved in 20 ml of 1% CH_3_COOH (pH =3.4–3.6) and aminoethylethanolamine (AEEA) (2.0 ml) was reacted at 100°C for 4 h to obtain compound 1. To adjacent the pH of compound 1, 1 M NaOH was added. An electrophilic substitution process in which the proton was replaced by the SO_3_H group was the primary mechanism of the reaction (Morrison and Boyd, 1997). Compound 1 was dissolved in dimethylformamide (DMF), chlorosulfonic acid (4.5 ml in 30 ml DMF) was fractionally added, and the mixture was stirred at room temperature for 5 h. It was neutralized with a 20% NaOH solution, and the precipitate of compound 2 was obtained, and reprecipitated with methanol twice (Martins et al., 2003). Compound 2 (2.5 g dissolved in acetic acid with ethanol) and benzaldehyde (1.0 ml) were mixed with ethanol and stirred for 8 h at 60°C. The white gel obtained indicates the formation of Schiff base compound 2a. A solution of 5% NaOH was used to precipitate the product, filtered, washed with ice-cold water, and add ethanol to remove any unreacted products. The final product was soluble in DMF. TLC was used to track the reaction’s progress. In TLC, the eluting solvents were hexane and ethylacetate (4:6). Compounds 2b–2j were synthesized using the technique outlined above.

### Synthesis of (2,3,4,6)-3-(benzylideneamino)-6-(((2-((2-hydroxyethyl)amino)ethyl)amino)methyl)-2,5-dimethoxytetrahydro-2H-pyran-4-yl sulfate (2a)

Yield: 65%;IR (KBr) (cm^−1^); 3,452 (NH, str), 3,280 (OH-str), 2,951 (CH-str Ar ring), 1,645 (N = CH, str), 1,383 (N-C, str), 1,326 (S = O), 972 (C-O-S, str). ^1^H NMR (DMSO-d_6_), δ (ppm): 8.20 (s, 1H, N = CH), 7.85–7.52 (m, 5H, Ph), 5.21–3.10 (m, 5H, CS-H), 3.68 (s, 1H, OH), 3.52–3.33 (s, 6H, OCH_3_ -CS), 3.45–2.70 (m, 4H, OH-(CH_2_)_2_), 2.80–2.54 (m, 2H, CH_2_-NH), 2.68–2.51 (m, 4H, N (CH_2_)_2_), 2.02 (s, 2H, NH); ^13^C NMR (DMSO-d_6_) δ (ppm): 163.8 (1C,N = CH), 136.5, 131.2, 129.3, 128.9 (6C, Ph), 112.3, 87.4, 71.5, 65.2, 60.7 (5C, CS), 61.7, 52.0 (2C, C-OH), 57.6, 55.7 (2C, OCH_3_-CS), 49.6, 49.0 (2C, N-C in amine), 48.4 (1C, N-CH).

### Synthesis of (2,3,4,6)-3-((4-chlorobenzylidene)amino)-6-(((2-((2-hydroxyethyl) amino)ethyl) amino)methyl)-2,5-dimethoxytetrahydro-2H-pyran-4-ylsulfate (2b)

Yield: 68%; IR (KBr) (cm^−1^); 3,454 (NH, str), 3,286 (OH-str), 2,954 (CH-str Ar ring), 1,641 (N = CH, str), 1,378 (N-C, str), 1,320 (S = O), 972 (C-O-S, str), 544 (C-Cl). ^1^H NMR (DMSO-d_6_), δ (ppm): 8.18 (s, 1H, N=CH), 7.80–7.50 (m, 4H, Cl-Ph), 5.23–3.12 (m, 5H, CS-H), 3.66 (s, 1H, OH), 3.54–3.35 (s, 6H, OCH_3_ -CS), 3.47–2.72 (m, 4H, OH-(CH_2_)_2_), 2.82–2.56 (m, 2H, CH_2_-NH), 2.65–2.50 (m, 4H, N (CH_2_)_2_), 2.04 (s, 2H, NH); ^13^C NMR (DMSO-d_6_) δ (ppm): 163.4 (1C,N = CH), 136.8, 134.2, 130.3, 129.0 (6C,Cl- Ph), 112.5, 87.6, 71.7, 65.4, 60.9 (5C, CS), 61.9, 52.2 (2C, C-OH), 57.8, 55.9 (2C, OCH_3_-CS), 49.8, 49.2 (2C, N-C in amine), 48.6 (1C, N-CH).

### Synthesis of (2,3,4,6)-3-((4-fluorobenzylidene)amino)-6-(((2-((2-hydroxyethyl)amino)ethyl)amino)methyl)-2,5-dimethoxytetrahydro-2H-pyran-4-ylsulfate (2c)

Yield: 70%; IR (KBr) (cm^−1^); 3,450 (NH, str), 3,282 (OH-str), 2,945 (CH-str Ar ring), 1,643 (N = CH, str), 1,384 (N-C, str), 1,322 (S = O), 978 (C-O-S, str). ^1^H NMR (DMSO-d_6_), δ (ppm): 8.41 (s, 1H, N=CH), 7.85–7.38 (m, 4H, F-Ph), 5.20–3.08 (m, 5H, CS-H), 3.65 (s, 1H, OH), 3.50–3.31 (s, 6H, OCH_3_ -CS), 3.43–2.68 (m, 4H, OH-(CH_2_)_2_), 2.81–2.53 (m, 2H, CH_2_-NH), 2.66–2.52 (m, 4H, N (CH_2_)_2_), 2.03 (s, 2H, NH); ^13^C NMR (DMSO-d_6_) δ (ppm): 165.5, 132.2, 130.3, 115.9 (6C, F-Ph), 163.6 (1C,N = CH), 112.1, 87.3, 71.2, 65.0, 60.5 (5C, CS), 61.4, 52.1 (2C, C-OH), 57.4, 55.3 (2C, OCH_3_-CS), 49.7, 49.1 (2C, N-C in amine), 48.2 (1C, N-CH).

### Synthesis of (2,3,4,6)-3-((4-hydroxybenzylidene)amino)-6-(((2-((2-hydroxyethyl)amino)ethyl)amino)methyl)-2,5-dimethoxytetrahydro-2H-pyran-4-ylsulfate (2d)

Yield: 62%; IR (KBr) (cm^−1^); 3,458 (NH, str), 3,285 (OH-str), 2,956 (CH-str Ar ring), 1,643 (N = CH, str), 1,385 (N-C, str), 1,327 (S = O), 980 (C-O-S, str). ^1^H NMR (DMSO-d_6_), δ (ppm): 10.54 (s, 1H, OH-Ph), 8.61 (s, 1H, N = CH), 7.80–6.82 (m, 4H, OH-Ph), 5.22–3.11 (m, 5H, CS-H), 3.69 (s, 1H, OH), 3.53–3.34 (s, 6H, OCH_3_ -CS), 3.46–2.71 (m, 4H, OH-(CH_2_)_2_), 2.81–2.53 (m, 2H, CH_2_-NH), 2.69–2.52 (m, 4H, N (CH_2_)_2_), 2.03 (s, 2H, NH); ^13^C NMR (DMSO-d_6_) δ (ppm): 163.9 (1C,N = CH), 160.5, 130.2, 129.3, 116.9 (6C, OH-Ph), 112.2, 87.3, 71.4, 65.3, 60.6 (5C, CS), 61.6, 52.1 (2C, C-OH), 57.5, 55.6 (2C, OCH_3_-CS), 49.7, 49.1 (2C, N-C in amine), 48.3 (1C, N-CH).

### Synthesis of (2,4,5,6)-2-(((2-((2-hydroxyethyl)amino)ethyl)amino)methyl)-3,6-dimethoxy-5-((4-methoxybenzylidene)amino)tetrahydro-2H-pyran-4-yl sulfate (2e)

Yield: 67%; IR (KBr) (cm^−1^); 3,455 (NH, str), 3,287 (OH-str), 2,952 (CH-str Ar ring), 1,646 (N = CH, str), 1,386 (N-C, str), 1,328 (S = O), 982 (C-O-S, str). ^1^H NMR (DMSO-d_6_), δ (ppm): 8.45 (s, 1H, N=CH), 7.89–7.10 (m, 4H, OCH_3_-Ph), 5.25–3.14 (m, 5H, CS-H), 3.86 (s, 3H, OCH_3_), 3.70 (s, 1H, OH), 3.55–3.36 (s, 6H, OCH_3_ -CS), 3.48–2.74 (m, 4H, OH-(CH_2_)_2_), 2.84–2.57 (m, 2H, CH_2_-NH), 2.69–2.55 (m, 4H, N (CH_2_)_2_), 2.06 (s, 2H, NH); ^13^C NMR (DMSO-d_6_) δ (ppm): 163.4 (1C,N = CH), 162.5, 130.4, 128.3, 114.6 (6C, OCH_3_-Ph), 112.6, 87.7, 71.8, 65.5, 60.9 (5C, CS), 61.4, 52.3 (2C, C-OH), 57.9, 55.8 (2C, OCH_3_-CS), 55.4 (1C, OCH_3_-Ph), 49.9, 49.1 (2C, N-C in amine), 48.5 (1C, N-CH).

### Synthesis of (2,3,4,6)-3-((4-(dimethylamino)benzylidene)amino)-6-(((2-((2- hydroxyethyl) amino)ethyl)amino)methyl)-2,5-dimethoxytetrahydro-2H-pyran-4-ylsulfate (2f)

Yield: 72%; IR (KBr) (cm^−1^); 3,457 (NH, str), 3,286 (OH-str), 1944 (CH-str Ar ring), 1,645 (N = CH, str), 1,386 (N-C, str), 1,329 (S = O), 980 (C-O-S, str). ^1^H NMR (DMSO-d_6_), δ (ppm): 8.27 (s, 1H, N=CH), 7.55–6.86 (m, 4H, N(CH_3_)_2_-Ph), 5.25–3.14 (m, 5H, CS-H), 3.70 (s, 1H, OH), 3.56–3.37 (s, 6H, OCH_3_ -CS), 3.47–2.73 (m, 4H, OH-(CH_2_)_2_), 3.05 (s, 6H, N(CH_3_)_2_), 2.82–2.56 (m, 2H, CH_2_-NH), 2.64–2.50 (m, 4H, N (CH_2_)_2_), 2.06 (s, 2H, NH); ^13^C NMR (DMSO-d_6_) δ (ppm): 163.5 (1C,N = CH), 153.4, 126.0, 124.3, 112.0, 41.5 (8C, N(CH_3_)_2_-Ph), 112.5, 87.6, 71.7, 65.4, 60.3 (5C, CS), 61.8, 52.4 (2C, C-OH), 57.8, 55.4 (2C, OCH_3_-CS), 49.8, 49.1 (2C, N-C in amine), 48.6 (1C, N-CH).

### Synthesis of (2,4,5,6)-2-(((2-((2-hydroxyethyl)amino)ethyl)amino)methyl)-3,6-dimethoxy-5-((4-nitrobenzylidene)amino)tetrahydro-2H-pyran-4-yl sulfate (2g)

Yield: 76%;IR (KBr) (cm^−1^); 3,459 (NH, str), 3,285 (OH-str), 2,951 (CH-str Ar ring), 1,641 (N = CH, str), 1,474 (N-O, str), 1,385 (N-C, str), 1,321 (S = O), 973 (C-O-S, str). ^1^H NMR (DMSO-d_6_), δ (ppm): 8.26 (s, 1H, N = CH), 8.43–8.12 (m, 4H, NO_2_-Ph), 5.23–3.12 (m, 5H, CS-H), 3.64 (s, 1H, OH), 3.55–3.35 (s, 6H, OCH_3_ -CS), 3.47–2.73 (m, 4H, OH-(CH_2_)_2_), 2.82–2.53 (m, 2H, CH_2_-NH), 2.65–2.52 (m, 4H, N (CH_2_)_2_), 2.06 (s, 2H, NH); ^13^C NMR (DMSO-d_6_) δ (ppm): 163.3 (1C,N = CH), 150.5, 142.2, 130.3, 124.9 (6C, NO_2_-Ph), 112.4, 87.6, 71.3, 65.5, 60.4 (5C, CS), 61.3, 52.4 (2C, C-OH), 57.4, 55.3 (2C, OCH_3_-CS), 49.7, 49.2 (2C, N-C in amine), 48.5 (1C, N-CH).

### Synthesis of (2,3,4,6)-3-((4-hydroxy-3-methoxybenzylidene)amino)-6-(((2-((2-hydroxyethyl) amino)ethyl)amino)methyl)-2,5-dimethoxytetrahydro-2H-pyran-4-ylsulfate (2h)

Yield: 84%; IR (KBr) (cm^−1^); 3,456 (NH, str), 3,278 (OH-str), 2,949 (CH-str Ar ring), 1,647 (N = CH, str), 1,381 (N-C, str), 1,324 (S = O, str), 975 (C-O-S, str). ^1^H NMR (DMSO-d_6_), δ (ppm): 8.20 (s, 1H, N = CH), 7.55–6.93 (m, 3H, Vanillin), 5.37 (s, 1H, OH-Ph), 5.20–3.13 (m, 5H, CS-H), 3.63 (s, 1H, OH), 3.73 (s, 3H, OCH_3_), 3.50–3.31 (s, 6H, OCH_3_ -CS), 3.42–2.68 (m, 4H, OH-(CH_2_)_2_), 2.83–2.53 (m, 2H, CH_2_-NH), 2.65–2.52 (m, 4H, N (CH_2_)_2_), 2.07 (s, 2H, NH); ^13^C NMR (DMSO-d_6_) δ (ppm): 163.4 (1C, N = CH), 151.5, 149.2, 133.3, 123.0, 117.2, 112.5 (6C, Vanillin), 112.4, 87.1, 71.3, 65.0, 60.4 (5C, CS), 61.6, 52.2 (2C, C-OH), 57.5, 55.4 (2C, OCH_3_-CS), 56.5 (1C, OCH_3_), 49.7, 49.1 (2C, N-C in amine), 48.2 (1C, N-CH).

### Synthesis of (2,3,4,6)-3-((2-hydroxybenzylidene)amino)-6-(((2-((2- hydroxyethyl) amino)ethyl) amino)methyl)-2,5-dimethoxytetrahydro-2H-pyran-4-yl sulfate (2i)

Yield: 73%; IR (KBr) (cm^−1^); 3,458 (NH, str), 3,284 (OH-str), 2,950 (CH-str Ar ring), 1,642 (N = CH, str), 1,386 (N-C, str), 1,326 (S = O), 989 (C-O-S, str). ^1^H NMR (DMSO-d_6_), δ (ppm): 8.27 (s, 1H, N = CH), 7.68–7.03 (m, 4H, Ph), 5.45 (s, 1H, OH-Ph), 5.24–3.13 (m, 5H, CS-H), 3.65 (s, 1H, OH), 3.54–3.35 (s, 6H, OCH_3_ -CS), 3.43–2.74 (m, 4H, OH-(CH_2_)_2_), 2.83–2.55 (m, 2H, CH_2_-NH), 2.63–2.52 (m, 4H, N (CH_2_)_2_), 2.06 (s, 2H, NH); ^13^C NMR (DMSO-d_6_) δ (ppm): 163.6 (1C,N = CH), 161.5, 132.6, 132.0, 124.9, 124.0 (6C, Ph), 112.1, 87.2, 71.5, 65.3, 60.4 (5C, CS), 61.5, 52.6 (2C, C-OH), 57.7, 55.8 (2C, OCH_3_-CS), 49.9, 49.0 (2C, N-C in amine), 48.5 (1C, N-CH).

### Synthesis of (2,3,4,6)-3-((furan-2-ylmethylene)amino)-6-(((2-((2- hydroxyethyl)amino)ethyl) amino)methyl)-2,5-dimethoxytetrahydro-2H-pyran-4-yl sulfate (2j)

Yield: 68%;IR (KBr) (cm^−1^); 3,458 (NH, str), 3,279 (OH-str), 2,957 (CH-str Ar ring), 1,644 (N = CH, str), 1,383 (N-C, str), 1,321 (S = O), 812 (C-O-S, str). ^1^H NMR (DMSO-d_6_), δ (ppm): 8.25 (s, 1H, N = CH), 7.72–6.50 (m, 3H, furfural), 5.26–3.17 (m, 5H, CS-H), 3.68 (s, 1H, OH), 3.57–3.34 (s, 6H, OCH_3_ -CS), 3.43–2.72 (m, 4H, OH-(CH_2_)_2_), 2.81–2.55 (m, 2H, CH_2_-NH), 2.62–2.53 (m, 4H, N (CH_2_)_2_), 2.05 (s, 2H, NH); ^13^C NMR (DMSO-d_6_) δ (ppm): 163.2 (1C,N = CH), 149.6, 144.2, 118.3, 112.9 (4C, furfural), 112.9, 87.8, 71.7, 65.6, 60.5 (5C, CS), 61.4, 52.3 (2C, C-OH), 57.2, 55.1 (2C, OCH_3_-CS), 49.8, 49.1 (2C, N-C in amine), 48.6 (1C, N-CH).

## Biological Screening

### Cytotoxic Activity

The cytotoxic activity of the newly synthesized compounds (2a-2j) was tested using a previously described method (Scudiere et al., 1988; Surendra Kumar et al., 2017). Compounds 2a-2j was tested in the MCF-7 cell line for 48 h using a single dose of primary anticancer assay at a concentration of 100 µm (MTT anticancer assay). MCF-7 breast cell line was employed in this study. The MCF-7 cell line was pre-incubated on a micro titer plate in this technique. Each test’s results are expressed as a percentage of the treated cells’ growth relative to the untreated control cells. Compounds with anticancer activity were those that reduced the proliferation of the cell lines by 30% or less. 0.1 ml of the cell suspension (containing 5 × 10^6^ cells/100 µL)and 0.1 ml of the test solution (6.25–100 µg in 1% DMSO such that the final concentration of DMSO in media was less than 1%) were added to the 9 well plates and incubated at 37°C for 72 h in a 5 percent CO_2_ incubator. The control wells included 1 percent DMSO and cell suspension, while the blank contained simply cell suspension. After 72 h, 20 µL of MTT were added and left in the CO_2_ incubator for 2 h before adding 100 µL of propanol. To protect the plate from light, it was wrapped in aluminum foil. The 9 well plates were then shaken for 10–25 min in a rotary shaker. The 9 well plates were processed on an ELISA reader for absorbance at 562 nm after 10–20 min.

### Molecular Docking

The binding abilities and interaction between compound 2a-2j, Doxorubicin, and the Human microsomal cytochrome P450 2A6 complexed with Methoxsalen (PDB ID: 1Z11) protein were investigated using Autodock vina 1.1.2 (Trott and Olson, 2009). To discover the target protein, the Protein Data Bank (http://www.rcsb.org) was used (PDB ID: 1Z11). The 3D structures of the compounds 2a-2j and Doxorubicin were created using Chem3D Pro 12.0 and Chem Draw Ultra 12.0. The AutoDock Tools 1.5.6 application suite was used to build the input files for Autodock Vina. The grid co-ordinates for the target protein were observed to be 56.316, 77.155, 60.326, with size x,y,z: 24, 20, 20 and 1.0 spacing. The interpretation of exhaustiveness has been assigned the number eight. Other Vina docking parameters are enabled by default and are not listed. The compound with the lowest binding affinity value obtained the highest score, which was determined visually using the software Discovery Studio 2019.

## Result and Discussion

**SCHEME 1:**
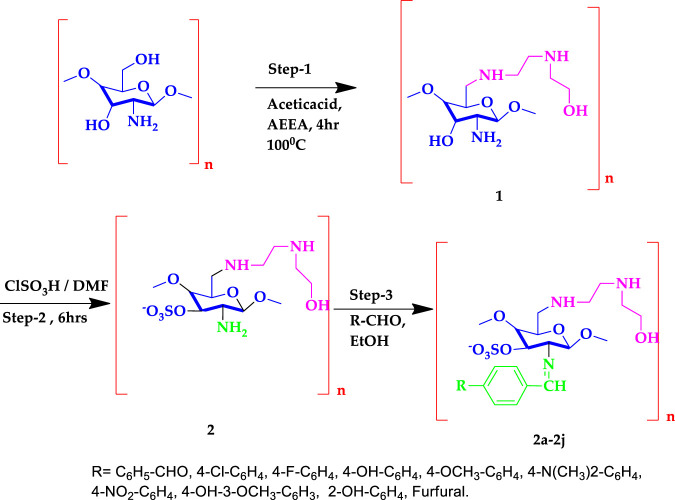
The synthesized Schiff base of sulfonated chitosan derivatives 2a-2j.

The novel chitosan compounds were established by ^1^H NMR, and ^13^C NMR, as presented in the Supplementary Materials. The FT-IR spectra presented absorption bands (2a–2j) at 3,450–3,458, 3,280–3,289, 3,050–3,057, 2,928, 1,645, 1,592, 1,474, 1,420, 1,242 and 815 cm^−1^, confirming the NH, OH, Ar-H, CH, N = CH, NH_2_, (N-O), N-C, (S = O), and (C-O-S) groups. The absorption bands of other compounds are summarized in the Experimental section. The ^1^H NMR spectra (2a–2j) show shifts in the chemical values at 8.18–8.45, 7.52–7.85, 6.50–7.72,3.64–3.70, 3.33–3.52, 3.10–5.21, 2.72–3.43, 2.54–2.80, 2.51–2.68and 2.02–2.07 ppm, confirming the presences of N=CH, Ph-CH, furfural, CH_2_-OH, OCH_3_, CS-H, OH-(CH_2_)_2_,CH_2_-NH, N(CH_2_)_2_, and NH protons, respectively. The ^1^H NMR spectra of other compounds are summarized in the Experimental section. The ^13^C NMR (2a–2j) showed signals at 163.2–165.5, 128.9–136.5, 112.9–149.6, 60.7–112.3, 52.0–61.7, 55.7–57.6, and 48.3–49.0 ppm, confirming the presence of carbon atom in N = CH, Ph, furfural, CS, C-OH, OCH_3_, N-C, and N-CH. The shift in chemical values of other compounds are given in the experimental section. Compound 2 (2.5 g dissolved in acetic acid with ethanol) and 4-chlorobenzaldehyde (1.2 ml) were mixed with ethanol and stirred for 8 h at 60°C. The light-yellow gel obtained indicates the formation of compound 2b. A solution of 5% NaOH was used to precipitate the product, was filtered, and was washed with ice-cold water and ethanol to remove any unreacted products. The final product was soluble in DMF. In this synthesize of chitosan derivatives without using any catalyst, we obtained better yields with in a shorter reaction time. The progress of the reaction was monitored by TLC. The same procedure was followed by the synthesis of other sulfonated chitosan derivatives 2c-2j.The synthesized compounds 2a-2j were assessed for their cytotoxic activities against the MCF-7 cancer cell line and against the normal cell lines breast cancer cell MCF-10A and lung cell MRC-5 using an MTT assay, and doxorubicin was used as a standard. Compound 2 h was found to be highly active, with a GI_50_ value of 0.02 µM for MCF-7 compared with other compounds. The synthesized chitosan derivatives showed less cytotoxicity in normal cell lines MCF-10A and MRC-5, with IC_50_ > 100 g/ml, indicating that their use is safe. Scheme 1 illustrates the synthesis of sulfonated chitosan derivatives.

### XRD or X-Ray Diffraction study

**FIGURE 2:**
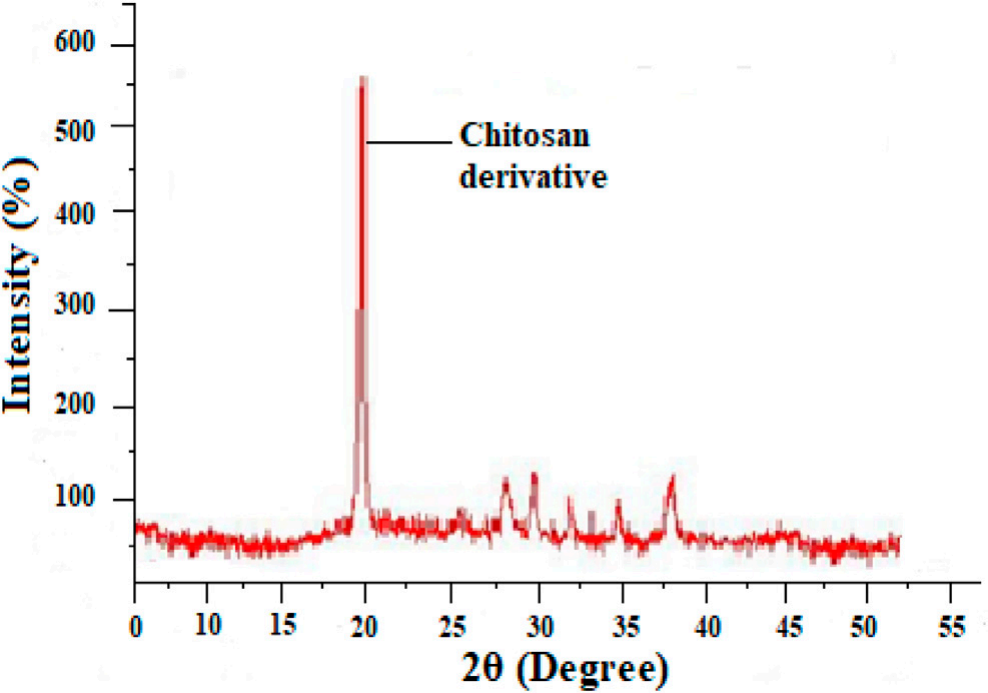
X-ray diffraction study of chitosan derivative.

An X-Ray diffraction investigation of chitosan derivatives are shown in Figure 2 (Kumar et al., 2009) it shows modest peaks for chitosan derivatives at 2θ of 20°. In chitosan derivatives the very sharp peaks at 20° became narrow, it indicates that chitosan derivative has moral compatibility and good development of porous xerogel network. According to the XRD results the chitosan derivative is amorphous in nature.

### FTIR spectroscopy of highly Active compound 2h

**FIGURE 3:**
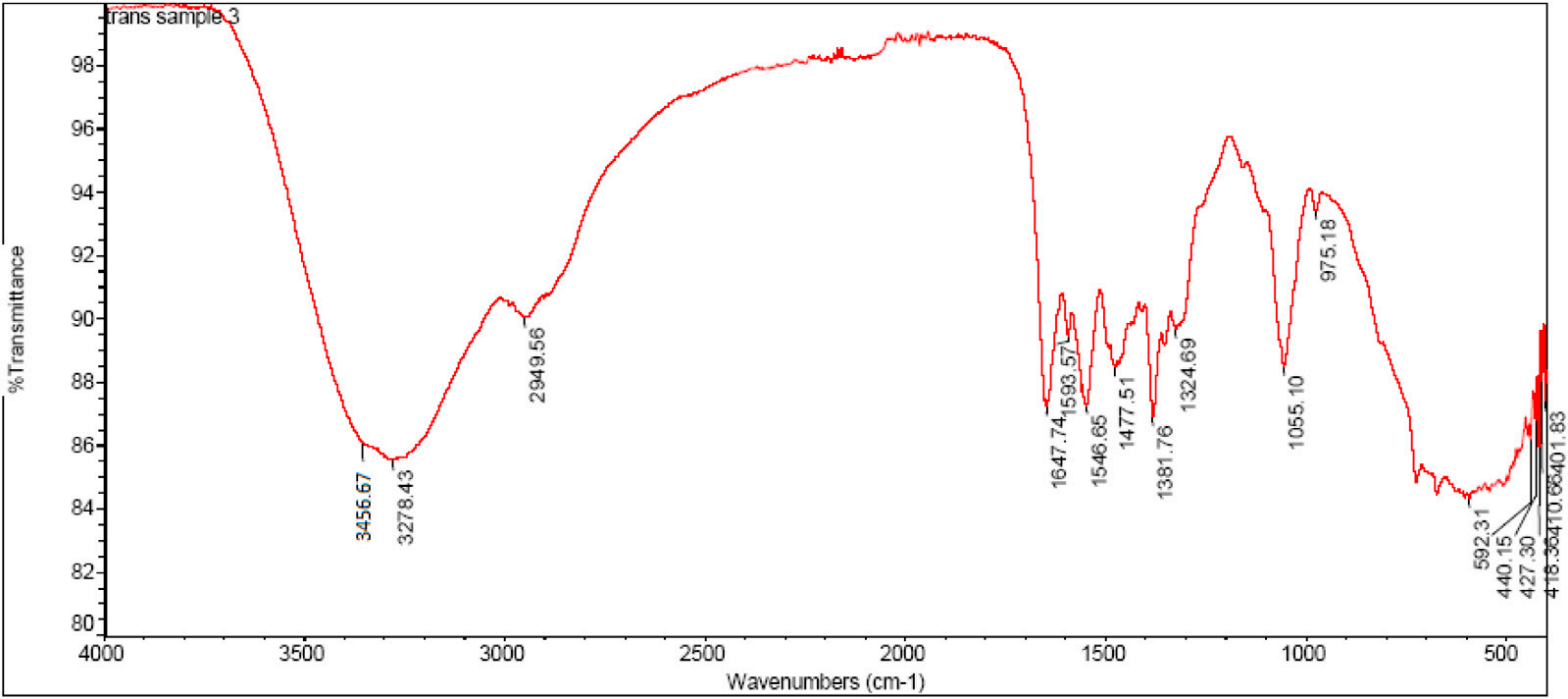
FTIR Spectrum of compound-2h.

In FTIR spectra of chitosan NH peak was observed at 3456cm^−1^which can be assigned to stretching vibration of amino group, another peak observed at 3,278 cm^−1^can be attributed to hydroxyl group (OH,str). Similarly the stretching vibration of carbon and hydrogen in aromatic ring observed at 2,949 cm^−1^. The sharp characteristic peak at 1647cm^−1^can be attributed to stretching vibration of imine linkage (N = CH, str). In the chitosan-Schiffbase the stretching vibration of N-C observed at 1381cm^−1^ can be assigned to (N-C, str), another sharp peak at 1324cm^−1^ can be attributed to stretching vibration of (S=O, str), and C-O-S can be observed as a stretching vibration of 975cm^−1^. The IR spectrum of highly active compound are shown in Figure 3.

### Scanning Electron Microscopy

**FIGURE 4:**
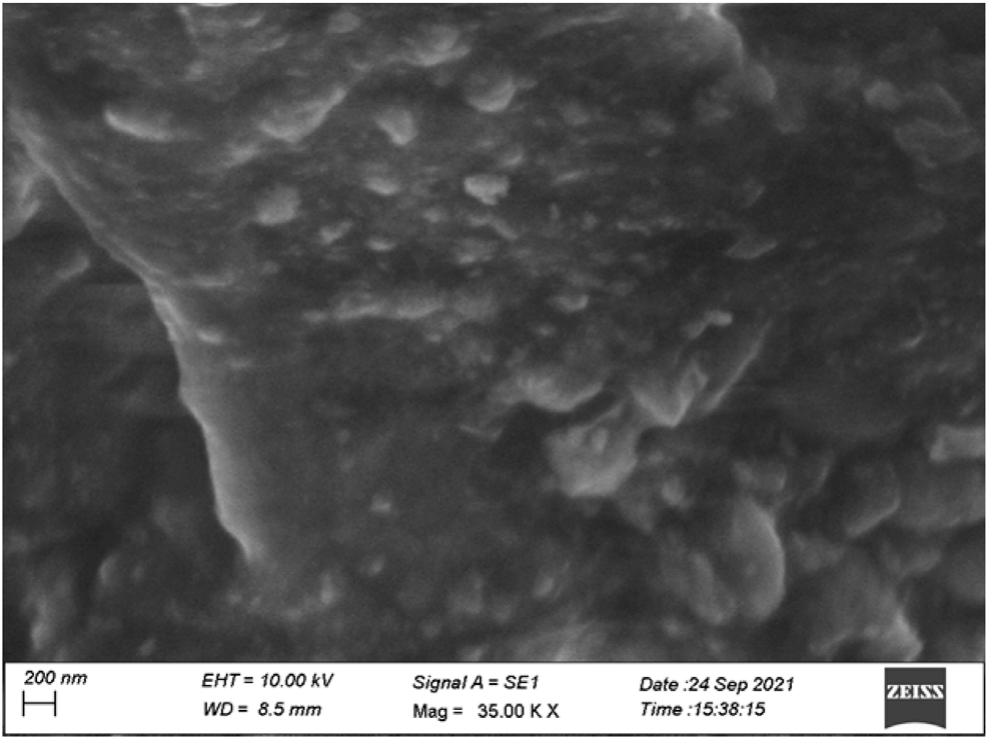
SEM image of chitosan derivative.

One of the most effective techniques for studying surface phenomena of prepared materials is the scanning electron microscope (SEM). The morphology of schiffbase of sulfonated chitosan derivative 2 h is the particles with sizes of 200 nm. These observations show that the microstructures of chitosan changed after chemical modification. The SEM image of chitosan analogues are shown in Figure 4.

### Cytotoxic Activity

The newly synthesized Schiff base of sulfonated chitosan derivatives (2a-2j) were screened for their cytotoxic activity using MTT anticancer assay (dose of 100 μM at 48 h) against MCF-7 cancer cell line, andalso evaluated for their possible cytotoxicity in breast cancer cell (MCF-10A), and lung cells (MRC-5) by employing MTT assay. The assay results suggested that these compounds did not significantly affect normal breast, lung cells’ growth (As most of the compound’s IC50 values are >100). The Table 1 shows the growth inhibitor concentration (GI_50_), total growth of inhibition (TGI), and lethal concentration (LC50) values. Moreover the compound 2 h (GI_50_ = 0.02 µM) was relatively more active than standard doxorubicin and other compounds. The *in vitro* cytotoxicity of Schiff base of sulfonated chitosan derivatives of normal cell lines are given in Table 2. Finally, compound 2 h can be used as a lead compound to further develop more effective drugs for the MCF-7 (breast) cancer cell line.

**TABLE 1:** Anticancer activity of compounds (µM) (2a-2j).

Compounds	MCF-7 cell line		
	GI_50_ (µM)	TGI (µM)	LC_50_ (µM)
2a	03.3 ± 0.23	07.3 ± 1.15	07.5 ± 0.03
2b	0.05 ± 0.15	0.65 ± 2.45	0.89 ± 0.28
2c	0.20 ± 0.82	0.50 ± 1.50	0.75 ± 0.35
2d	0.10 ± 0.74	0.70 ± 0.68	1.50 ± 1.40
2e	0.05 ± 0.33	0.06 ± 1.32	0.79 ± 1.25
2f	0.65 ± 0.10	11.0 ± 1.62	20.3 ± 0.24
2g	0.43 ± 0.90	0.75 ± 1.53	15.6 ± 0.72
2h	0.02 ± 0.24	0.15 ± 0.19	0.72 ± 0.31
2i	0.07 ± 0.56	0.60 ± 0.41	2.25 ± 0.31
2j	0.04 ± 0.50	0.29 ± 0.72	9.12 ± 1.21
Doxorubicin	0.04 ± 0.75	0.25 ± 0.23	0.80 ± 0.46

Data represent the mean ± standard deviation (SD) of the mean values of three separate experiments.

**TABLE 2:** *In vitro* cytotoxicity of chitosan derivatives (2a-2j) on normal cells.

Compd. No	MCF-10A	MRC5
—	IC50 (µM)	IC50 (µM)
2a	65.62	60.53
2b	72.04	75.35
2c	71.51	73.03
2d	54.16	50.37
2e	62.08	67.59
2f	56.80	51.11
2g	74.62	70.23
2h	86.5	80.84
2i	69.04	58.95
2j	52.46	61.27

Each compound was tested in triplicate. All error bars represent mean ± SD from three independent experiments.

### Docking studies

**FIGURE 5:**
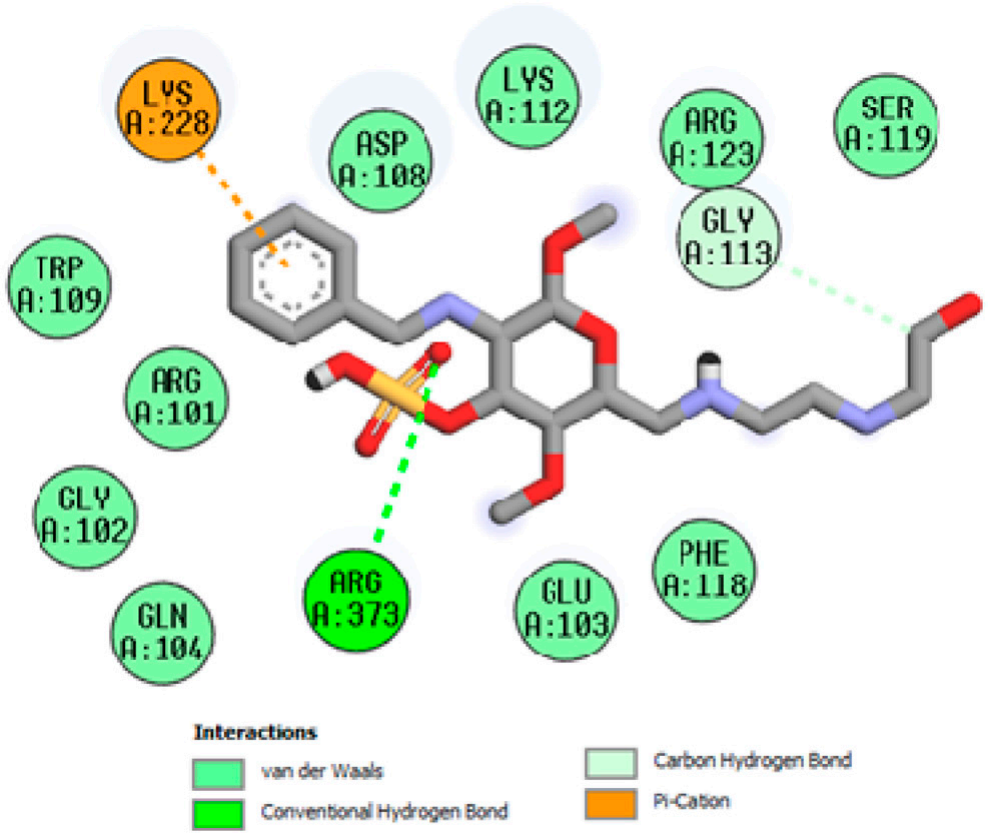
Interaction of compound 2a in the binding pocket of 1Z11 receptor.

**FIGURE 6:**
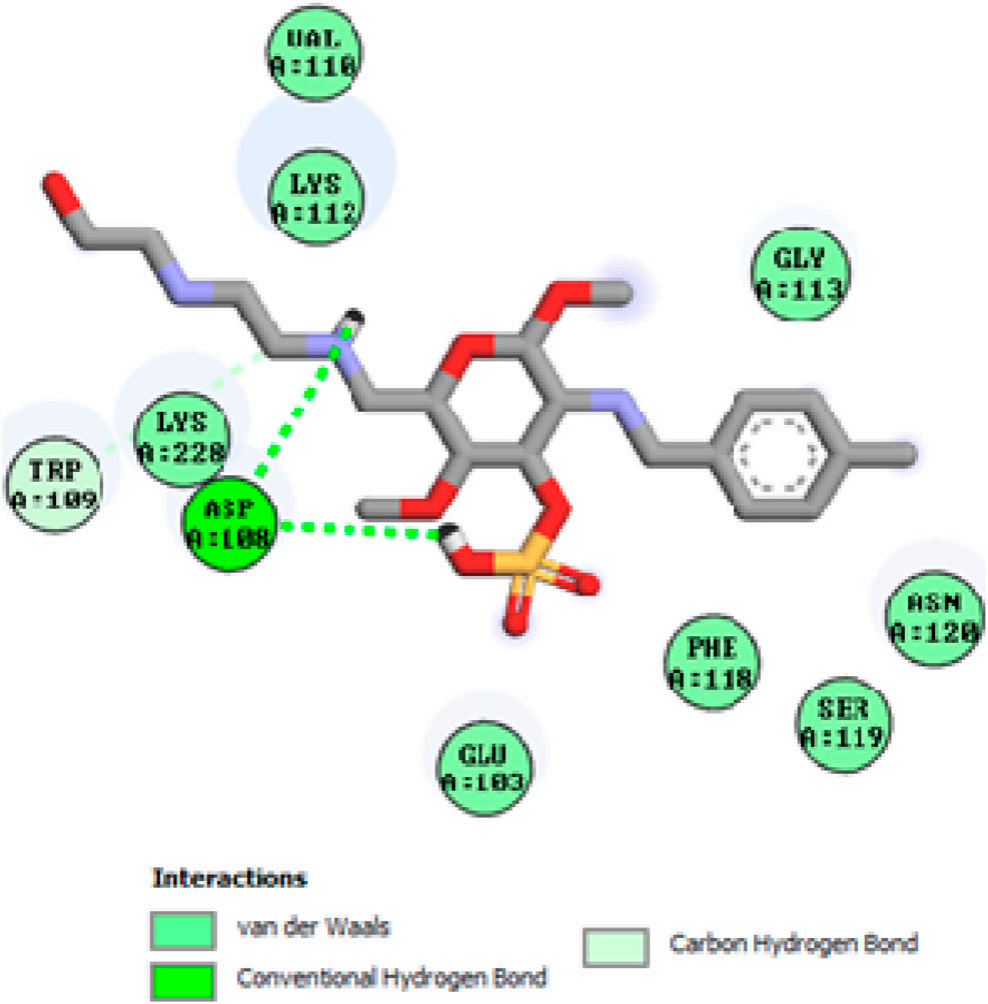
Interaction of compound 2b in the binding pocket of 1Z11 receptor.

**FIGURE 7:**
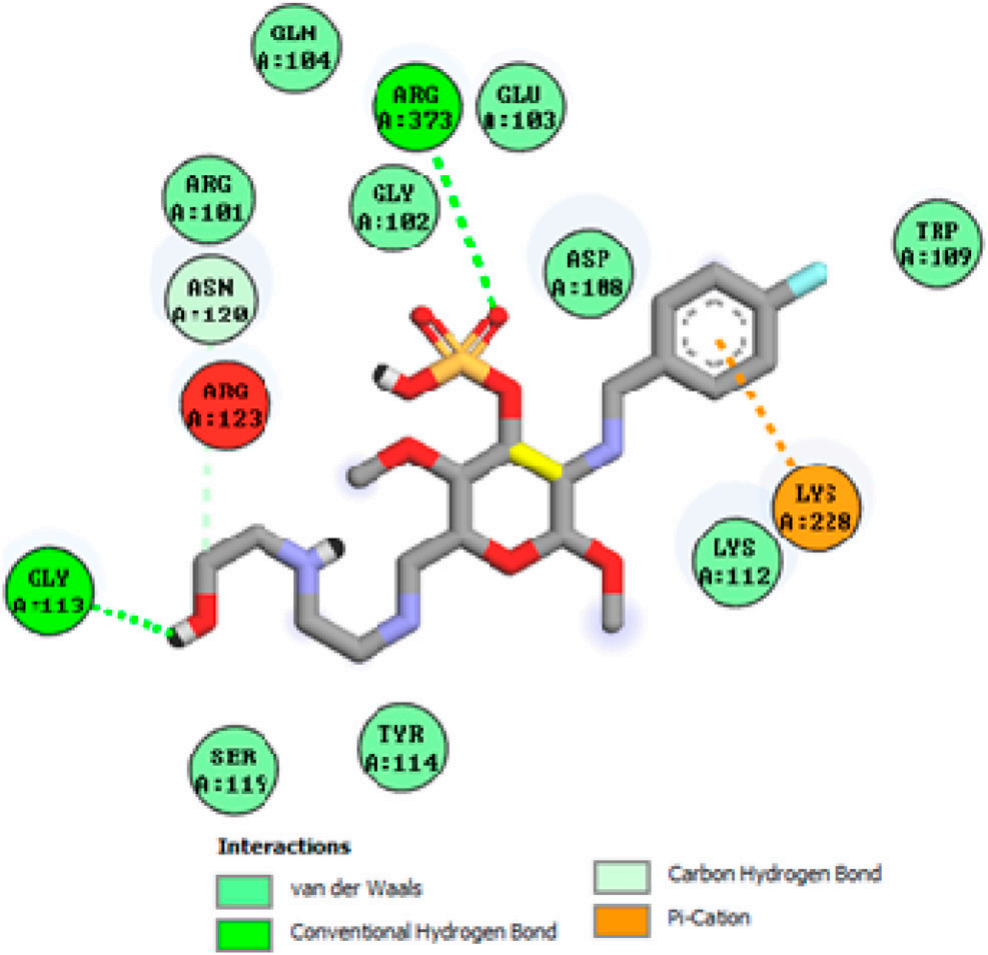
Interaction of compound 2c in the binding pocket of 1Z11 receptor.

**FIGURE 8:**
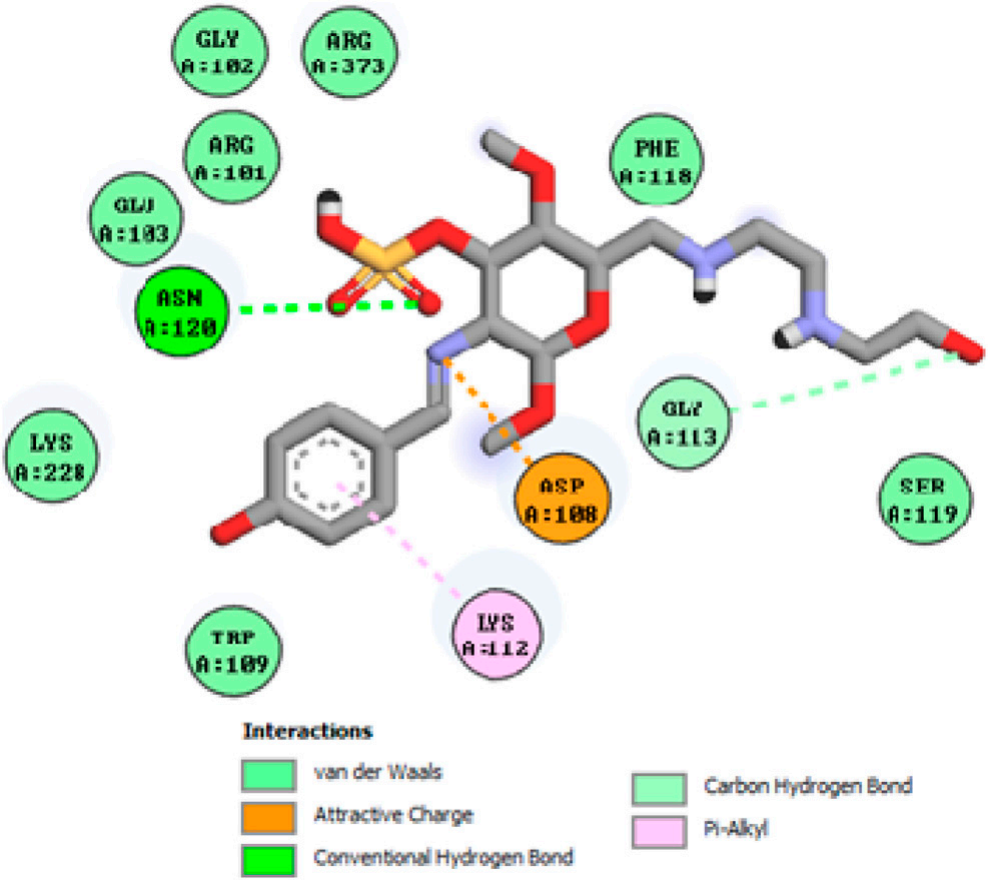
Interaction of compound 2d in the binding pocket of 1Z11 receptor.

**FIGURE 9:**
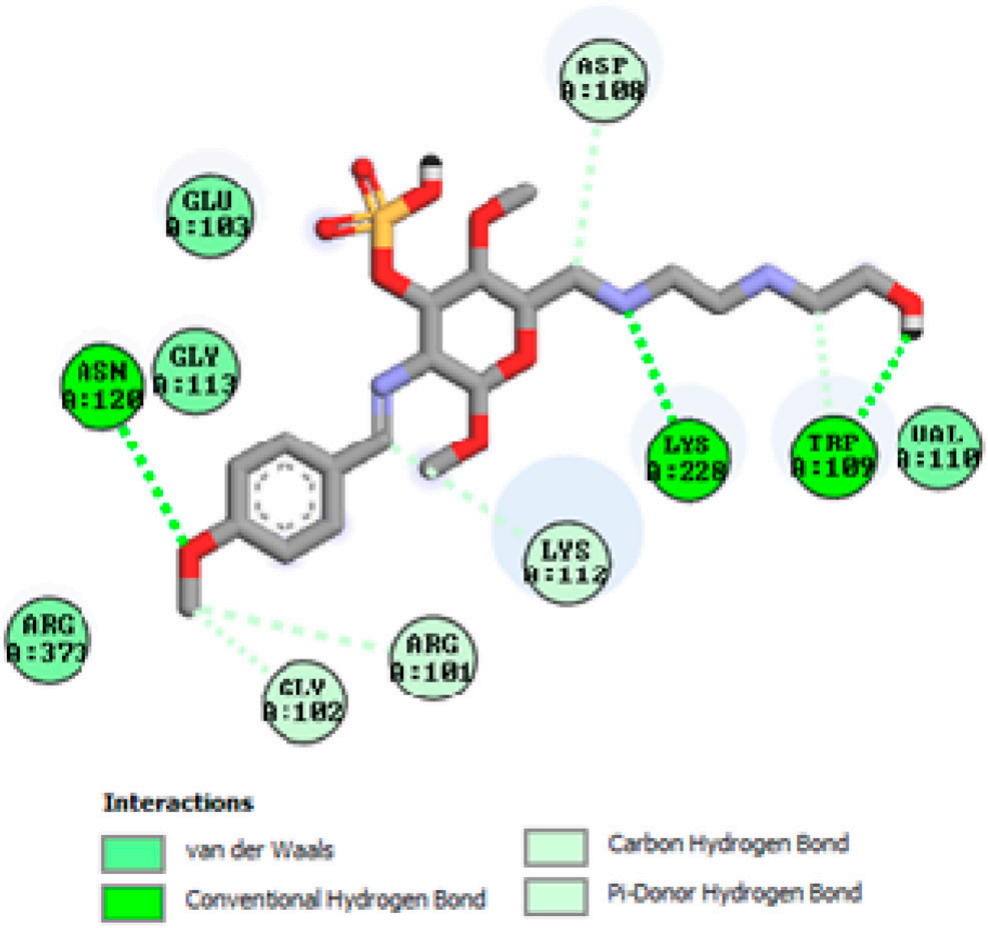
Interaction of compound 2e in the binding pocket of 1Z11 receptor.

**FIGURE 10:**
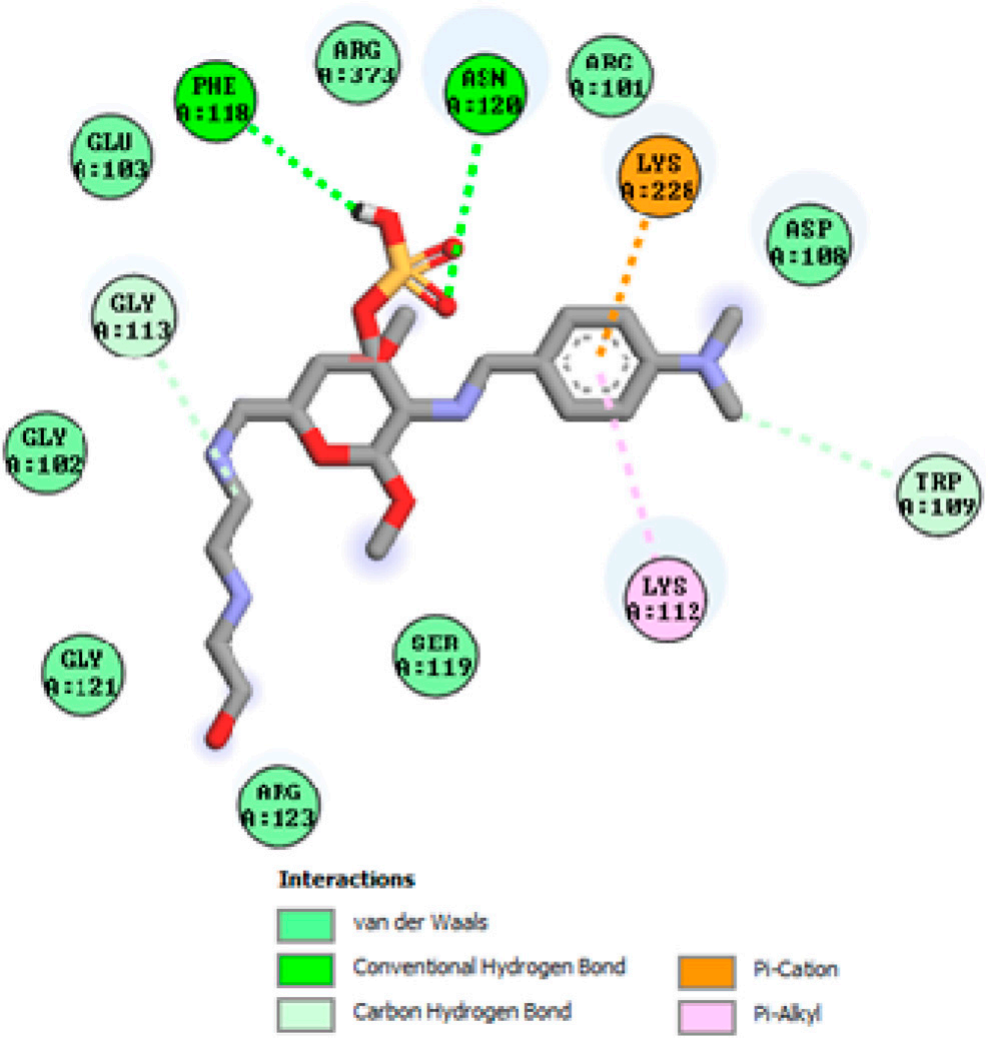
Interaction of compound 2f in the binding pocket of 1Z11 receptor.

**FIGURE 11:**
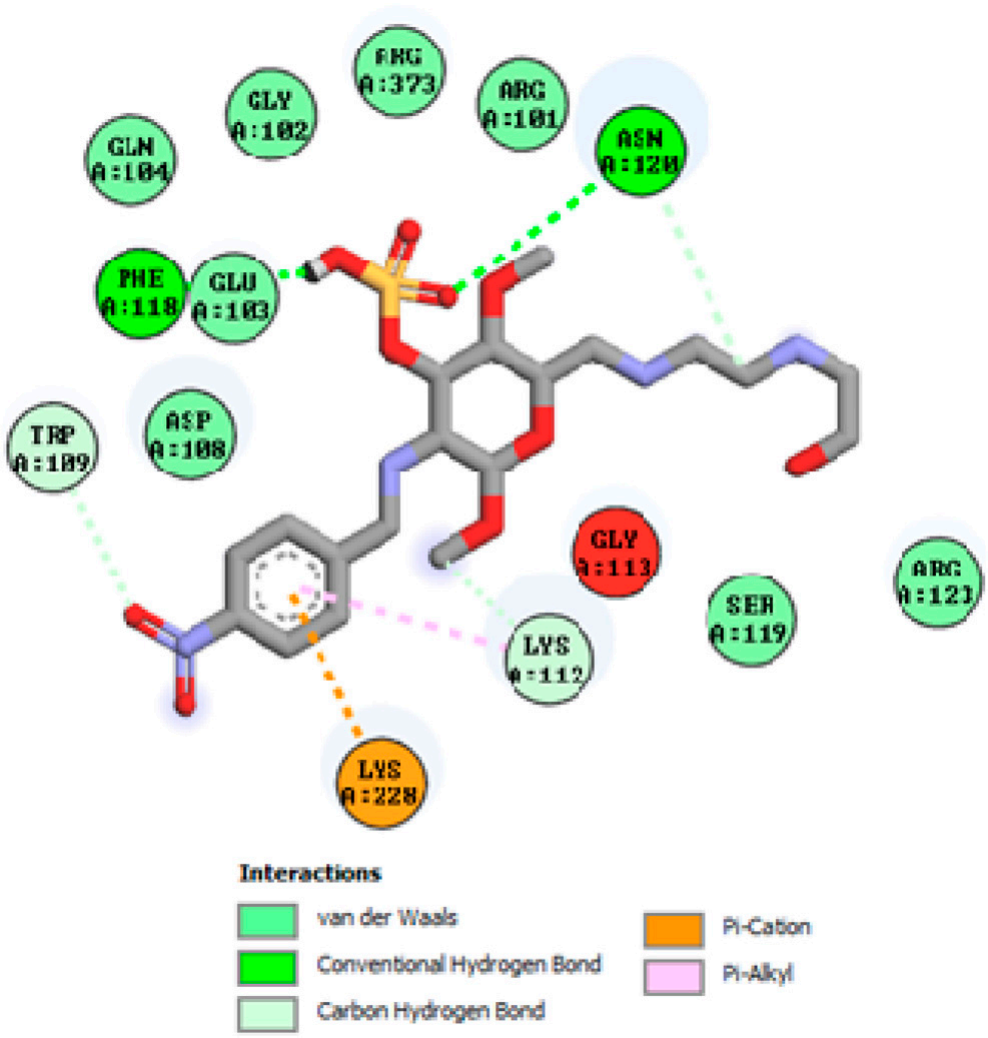
Interaction of compound 2g in the binding pocket of 1Z11 receptor.

**FIGURE 12:**
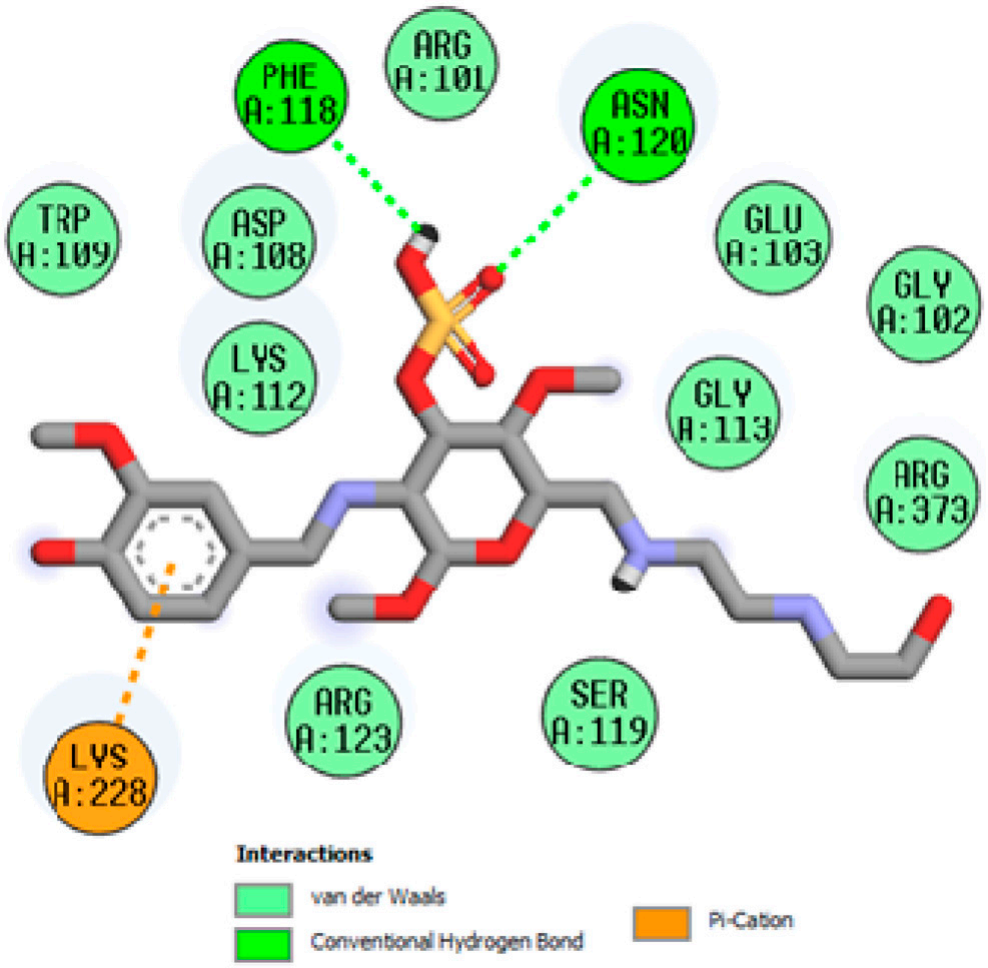
Interaction of compound 2h in the binding pocket of 1Z11 receptor.

**FIGURE 13:**
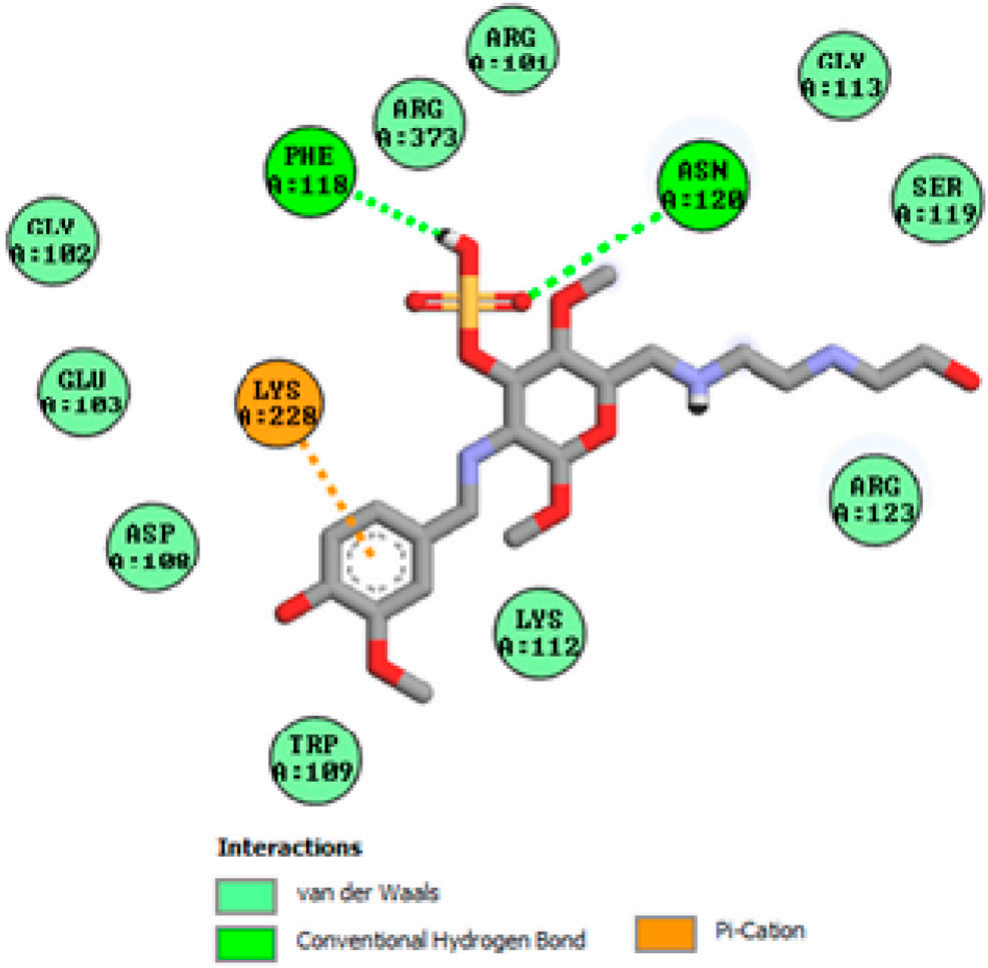
Interaction of compound 2i in the binding pocket of 1Z11 receptor.

**FIGURE 14:**
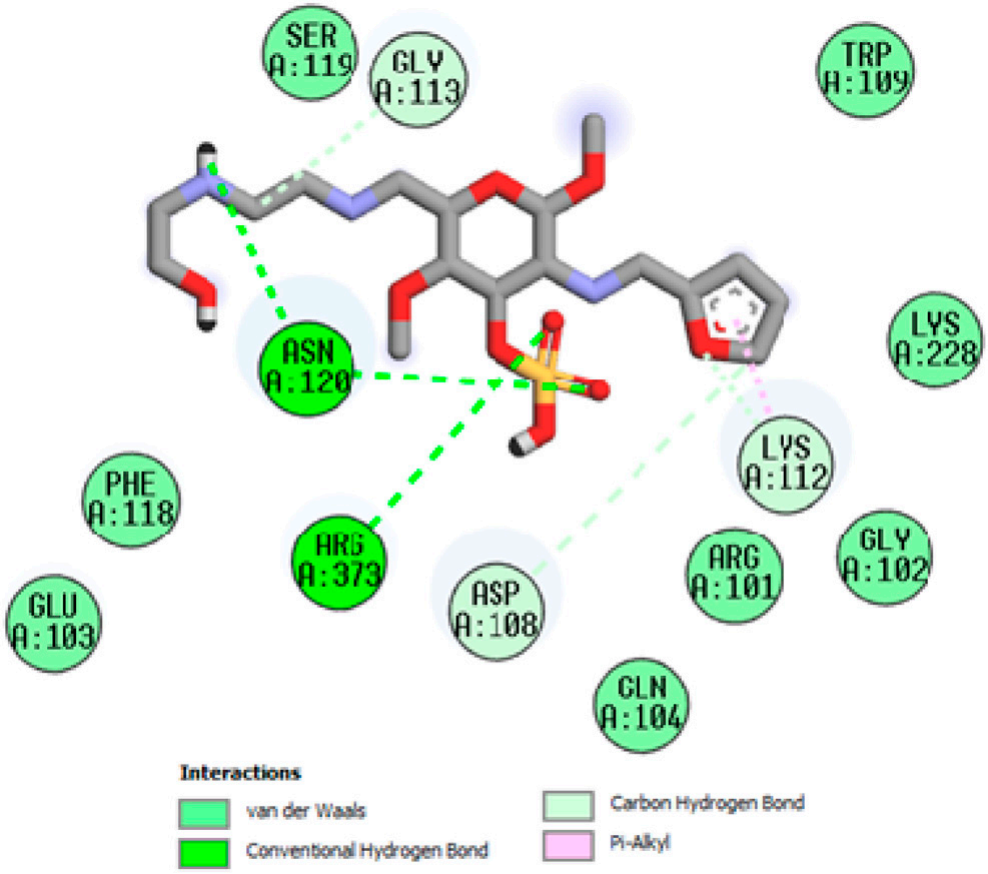
Interaction of compound 2j in the binding pocket of 1Z11 receptor.

**FIGURE 15:**
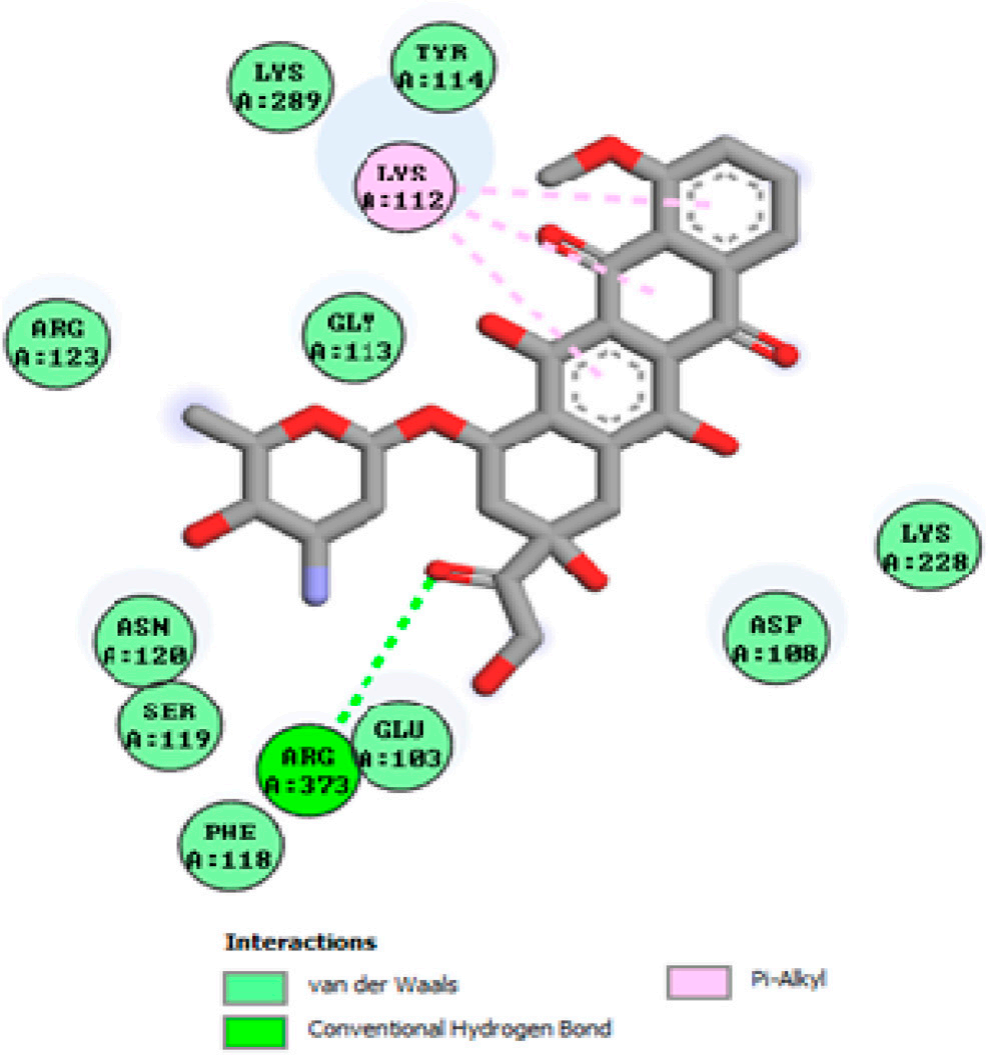
Interaction of compound Doxorubicin in the binding pocket of 1Z11 receptor.

To acquire a better understanding of the future evolution of biotic behavior, docking mock-ups were added. The docking impact of compounds 2a-2j and Doxorubicin regulation with protein 1Z11 was tested using the Autodock Vina software. Compound 2 h has a higher binding affinity for 1Z11 protein (−5.9 kcal/mol) than other compounds and a lower binding affinity for Doxorubicin (−5.3 kcal/mol). Compound 2 h makes two hydrogen bonds with the 1Z11 receptor. In the hydrogen bonding interaction, the amino acid residues Phe118 and Asn120 were active. In hydrophobic interaction, the amino acid residues Arg101, Gly102, Glu103, Asp108, Trp109, Lys112, Gly113, Ser119, Arg123, Lys228 and Arg373 were active. Doxorubicin regulates the 1Z11 receptor by forming one hydrogen bonds with it. In the hydrogen bonding interaction, the amino acid residues Arg373 were active. The hydrophobic interactions of Glu103, Asp108, Lys112, Gly113, Tyr114, Phe118, Ser119, Asn120, Arg123, Lys228 and Lys289 were observed. Figures 6, 7 show the hydrogen bonding and hydrophobic interactions of amino acid residues in the 1Z11 protein with the 2a-2j and Doxorubicin compounds, respectively. The results suggest that compound 2 h has a significant inhibitory effects for Doxorubicin regulation in the target protein. The docking interaction results are present in Table 3. All the docking images are given in Figures 5–15.

**TABLE 3:** Molecular docking interaction of compounds 2a-2j and Doxorubicin against protein 1Z11.

Compound	Human microsomal cytochrome P450 2A6 complexed with methoxsalen (PDB ID: 1Z11)		
	Binding affinity (kcal/mol)	No. of H-bonds	H-bonding residues
2a	−5.6	1	Arg373
2b	−5.0	2	Asp108
2c	−5.2	2	Gly113 and Arg373
2d	−4.8	1	Asn120
2e	−4.4	3	Trp109, Asn120 and Lys228
2f	−5.7	2	Phe118 and Asn120
2g	−4.8	2	Phe118 and Asn120
2h	−5.9	2	Phe118 and Asn120
2i	−4.8	2	Phe118 and Asn120
2j	−5.2	3	Asn120 and Arg373
Doxorubicin	−5.3	1	Arg373

### Structure Activity Relationship

**FIGURE 16:**
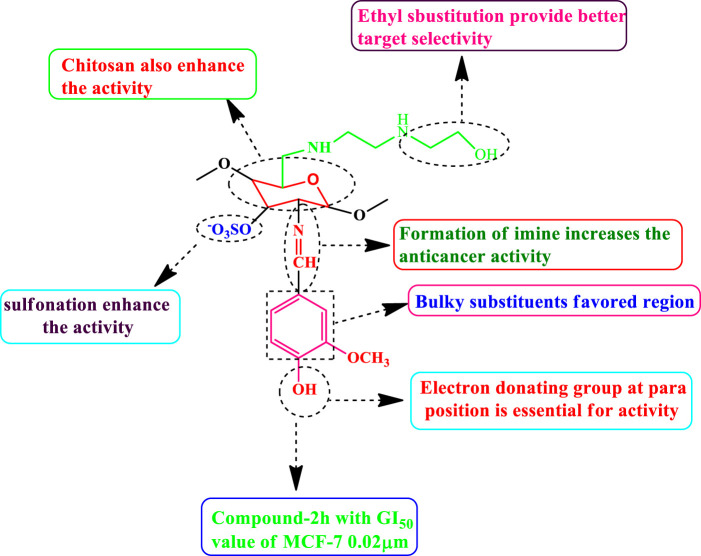
SAR of highly active compound.

The selected functional group plays important role in modifying the cytotoxic activity of compounds within a certain system can be identified via SAR analysis. Preliminary SARs might be analyzed using the cytotoxic activity data of the chitosan derivatives. Compound 2 h is the most effective (MCF-7, GI_50_ = 0.02 µM) control doxorubicin among the chitosan derivatives. Owing to the occurrence of chitosan moiety fused to a vanillin, it was revealed that the new compound acquires a strong cytotoxic effect against cancer cell types the presence of electron donating hydroxyl group in para position enhances the activity as well as sulfonation of chitosan also increases the anticancer activity. SAR of highly active compound present in Figure 16.

## Conclusion

In this paper, we synthesized chitosan derivatives of 2a-2j that were characterized via FTIR, NMR, and morphology study was carried out by XRD and SEM. The synthesized compounds 2a-2j were assessed for their cytotoxic activities against the MCF-7 cancer cell line, normal cell lines of breast cancer cell MCF-10A and lung cell MRC-5 using an MTT assay. Doxorubicin was used as a standard. Compound 2 h was found to be highly active, with a GI_50_ value of 0.02 µM for MCF-7 compared with other compounds. The synthesized chitosan derivatives showed less cytotoxicity in normal cell lines MCF-10A and MRC-5, with IC_50_ > 100 g/ml, indicating that their use is safe. Furthermore *In silico* molecular docking analysis against the 1Z11 receptor has been studied, compound 2 h has a higher binding affinity for 1Z11 protein (−5.9 kcal/mol) compared with other compounds and Doxorubicin (−5.3 kcal/mol). Finally, the compound 2 h has great activity, and therefore it will be researched for further anticancer drug.

## Data Availability

The datasets presented in this study can be found in online repositories. The names of the repository/repositories and accession number(s) can be found in the article/Supplementary Material.

## References

[B1] Adhikari H. S. Yadav P. N. (2018). Anticancer Activity of Chitosan, Chitosan Derivatives, and Their Mechanism of Action. Int. J. Biomater. 2018, 1–29. 10.1155/2018/2952085 PMC6332982 30693034

[B2] Ahmed S. Ikram S. (2016). Chitosan Based Scaffolds and Their Applications in Wound Healing. Achievements Life Sci. 10 (1), 27–37. 10.1016/j.als.2016.04.001

[B3] Ali I. Lone M. Al-Othman Z. Al-Warthan A. Sanagi M. (2015). Heterocyclic Scaffolds: Centrality in Anticancer Drug Development. Curr. Drug Targets 16 (7), 711–734. 10.2174/1389450116666150309115922 25751009

[B4] Ali I. Wani W. A. Saleem K. (2011). Cancer Scenario in India with Future Perspectives. Cancer Ther. 8 (1), 56–70.

[B5] Ali S. Sangi L. Kumar N. Kumar B. Khurshid Z. Zafar M. S. (2020). Evaluating Antibacterial and Surface Mechanical Properties of Chitosan Modified Dental Resin Composites. Technol. Health Care 28, 165–173. 10.3233/THC-181568 31594266

[B6] Andreadis C. Vahtsevanos K. Sidiras T. Thomaidis I. Antoniadis K. Mouratidou D. (2003). 5-Fluorouracil and Cisplatin in the Treatment of Advanced Oral Cancer. Oral Oncol. 39 (4), 380–385. 10.1016/S1368-8375(02)00141-0 12676258

[B7] Aranaz I. Harris R. Heras A. (2010). Chitosan Amphiphilic Derivatives. Chemistry and Applications. Curr. Org. Chem. 14 (3), 308–330. 10.2174/138527210790231919

[B8] Bakar L. M. Abdullah M. Z. Doolaanea A. A. Ichwan S. J. A. (2017). PLGA-chitosan Nanoparticle-Mediated Gene Delivery for Oral Cancer Treatment: a Brief Review. J. Phys. Conf. Ser. 884, 012117. 10.1088/1742-6596/884/1/012117

[B9] Bray F. Ferlay J. Soerjomataram I. Siegel R. L. Torre L. A. Jemal A. (2018). Global Cancer Statistics 2018: GLOBOCAN Estimates of Incidence and Mortality Worldwide for 36 Cancers in 185 Countries. CA: A Cancer J. Clinicians 68, 394–424. 10.3322/caac.21492 30207593

[B10] Bray F. Møller B. (2006). Predicting the Future burden of Cancer. Nat. Rev. Cancer 6 (1), 63–74. 10.1038/nrc1781 16372017

[B11] Chen G. Gong R. (2016). Study on Fluorouracil-Chitosan Nanoparticle Preparation and its Antitumor Effect. Saudi Pharm. J. 24, 250–253. 10.1016/j.jsps.2016.04.008 27275110 PMC4881156

[B12] Cheung R. Ng T. Wong J. Chan W. (2015). Chitosan: an Update on Potential Biomedical and Pharmaceutical Applications. Mar. Drugs 13 (8), 5156–5186. 10.3390/md13085156 26287217 PMC4557018

[B13] Husain S. Al-Samadani K. H. Najeeb S. Zafar M. S. Khurshid Z. Zohaib S. (2017). Chitosan Biomaterials for Current and Potential Dental Applications. Materials 10, 602. 10.3390/ma10060602 PMC5553419 28772963

[B14] Imran M. Sajwan M. Alsuwayt B. Asif M. (2020). Synthesis, Characterization and Anticoagulant Activity of Chitosan Derivatives. Saudi Pharm. J. 28, 25–32. 10.1016/j.jsps.2019.11.003 31920430 PMC6950966

[B15] Jiang M. Ouyang H. Ruan P. Zhao H. Pi Z. Huang S. (2011). Chitosan Derivatives Inhibit Cell Proliferation and Induce Apoptosis in Breast Cancer Cells. Anticancer Res. 31, 1321–1328. 21508382

[B16] Kim S. (2018). Competitive Biological Activities of Chitosan and its Derivatives: Antimicrobial, Antioxidant, Anticancer, and Anti-inflammatory Activities. Int. J. Polym. Sci. 2018, 1–13. 10.1155/2018/1708172

[B17] Kumar S. Dutta J. Dutta P. K. (2009). Preparation and Characterization of N-Heterocyclic Chitosan Derivative Based Gels for Biomedical Applications. Int. J. Biol. Macromolecules 45, 330–337. 10.1016/j.ijbiomac.2009.08.002 19665475

[B18] Kumirska J. Weinhold M. X. Thöming J. Stepnowski P. (2011). Biomedical Activity of Chitin/Chitosan Based Materials-Influence of Physicochemical Properties Apart from Molecular Weight and Degree of N-Acetylation. Polymers 3 (4), 1875–1901. 10.3390/polym3041875

[B19] Kuppusamy S. Karuppaiah J. (2013). Screening of Antiproliferative Effect of Chitosan on Tumor Growth and Metastasis in T24 Urinary Bladder Cancer Cell Line. Austrl-Asian J. ofCancer. 12 (3), 145–149.

[B20] Li Q. Li Q. Tan W. Zhang J. Guo Z. (2020). Phenolic-containing Chitosan Quaternary Ammonium Derivatives and Their Significantly Enhanced Antioxidant and Antitumor Properties. Carbohydr. Res. 498, 108169. 10.1016/j.carres.2020.108169 33059099

[B21] Li Z. Yang F. Yang R. (2015). Synthesis and Characterization of Chitosan Derivatives with Dual-Antibacterial Functional Groups. Int. J. Biol. Macromolecules 75, 378–387. 10.1016/j.ijbiomac.2015.01.056 25666853

[B22] Mahmood M. A. Madni A. Rehman M. Rahim M. A. Jabar A. (2019). Ionically Cross-Linked Chitosan Nanoparticles for Sustained Delivery of Docetaxel: Fabrication, Post-Formulation and Acute Oral Toxicity Evaluation. Int. J. Nanomedicine 14, 10035–10046. 10.2147/IJN.S232350 31908458 PMC6929931

[B23] Martins C. R. Ruggeri G. De Paoli M.-A. (2003). Synthesis in Pilot Plant Scale and Physical Properties of Sulfonated Polystyrene. J. Braz. Chem. Soc. 14, 797–802. 10.1590/S0103-50532003000500015

[B24] Morrison R. T. Boyd R. N. (1997). Organic Chemistry. Sixth ed. Hoboken, NJ, USA: Prentice-Hall, 517–527.

[B25] Muanprasat C. Chatsudthipong V. (2017). Chitosan Oligosaccharide: Biological Activities and Potential Therapeutic Applications. Pharmacol. Ther. 170, 80–97. 10.1016/j.pharmthera.2016.10.013 27773783

[B26] Pereira C. Leao M. Soares J. Bessa C. Saraiva L. (2012). New Therapeutic Strategies for Cancer and Neurodegeneration Emerging from Yeast Cell-Based Systems. Curr. Pharm. Des. 18 (27), 4223–4235. 10.2174/138161212802430422 22650181

[B27] Perni S. Prokopovich P. (2017). Poly-beta-amino-esters Nano-Vehicles Based Drug Delivery System for Cartilage. Nanomedicine: Nanotechnology, Biol. Med. 13, 539–548. 10.1016/j.nano.2016.10.001 PMC5339075 27746232

[B28] Qasim S. Zafar M. Najeeb S. Khurshid Z. Shah A. Husain S. (2018). Electrospinning of Chitosan-Based Solutions for Tissue Engineering and Regenerative Medicine. Int. J. Mol. Sci. 19, 407. 10.3390/ijms19020407 PMC5855629 29385727

[B29] Saeed R. M. Dmour I. Taha M. O. (2020). Stable Chitosan-Based Nanoparticles Using Polyphosphoric Acid or Hexametaphosphate for Tandem Ionotropic/Covalent Crosslinking and Subsequent Investigation as Novel Vehicles for Drug Delivery. Front. Bioeng. Biotechnol. 8, 4. 10.3389/fbioe.2020.00004 32039190 PMC6993129

[B30] Sarwar M. S. Huang Q. Ghaffar A. Abid M. A. Zafar M. S. Khurshid Z. (2020). A Smart Drug Delivery System Based on Biodegradable Chitosan/Poly(allylamine Hydrochloride) Blend Films. Pharmaceutics 12, 131. 10.3390/pharmaceutics12020131 PMC7076397 32033138

[B31] Scudiere D. A. Shoemaker R. H. Paul K. D. Monks A. Tierney S. Nofziger T. H. (1988). Evaluation of a Soluble Tetrazolium/Formazan Assay for Cell Growth and Drug Sensitivity in Culture Using Human and Other Tumor Cell Lines. Cancer Res. 48, 4827–4833. 3409223

[B32] Srivastava S. Koay E. J. Borowsky A. D. De Marzo A. M. Ghosh S. Wagner P. D. (2019). Cancer Overdiagnosis: a Biological challenge and Clinical Dilemma. Nat. Rev. Cancer 19, 349–358. 10.1038/s41568-019-0142-8 31024081 PMC8819710

[B33] Surendra Kumar R. Moydeen M. Al-Deyab S. S. Manilal A. Idhayadhulla A. (2017). Synthesis of New Morpholine - Connected Pyrazolidine Derivatives and Their Antimicrobial, Antioxidant, and Cytotoxic Activities. Bioorg. Med. Chem. Lett. 27, 66–71. 10.1016/j.bmcl.2016.11.032 27889456

[B34] Taher F. A. Ibrahim S. A. El-Aziz A. A. Abou El-Nour M. F. El-Sheikh M. A. El-Husseiny N. (2019). Anti-Proliferative Effect of Chitosan Nanoparticles (Extracted from Crayfish Procambarus Clarkii, Crustacea: Cambaridae) against MDA-MB-231 and SK-BR-3 Human Breast Cancer Cell Lines. Int. J. Biol. Macromolecules 126, 478–487. 10.1016/j.ijbiomac.2018.12.151 30572045

[B35] Trott O. Olson A. J. (2009). AutoDock Vina: Improving the Speed and Accuracy of Docking with a New Scoring Function, Efficient Optimization, and Multithreading. J. Comput. Chem. 31, 455. 10.1002/jcc.21334 PMC3041641 19499576

[B36] Wang P. He H. Cai R. Tao G. Yang M. Zuo H. (2019). Cross-linking of Dialdehyde Carboxymethyl Cellulose with Silk Sericin to Reinforce Sericin Film for Potential Biomedical Application. Carbohydr. Polym. 212, 403–411. 10.1016/j.carbpol.2019.02.069 30832874

[B37] Wimardhani Y. S. F. Suniarti D. J. Freisleben H. Wanandi S. I. C. Siregar N. Ikeda M.-A. (2014). Chitosan Exerts Anticancer Activity through Induction of Apoptosis and Cell Cycle Arrest in Oral Cancer Cells. J. Oral Sci. 56 (2), 119–126. 10.2334/josnusd.56.119 24930748

[B38] Zhang M. Li X. H. Gong Y. D. Zhao N. M. Zhang X. F. (2002). Properties and Biocompatibility of Chitosan Films Modified by Blending with PEG. Biomaterials 23 (13), 2641–2648. 10.1016/S0142-9612(01)00403-3 12059013

[B39] Zhang Y. Sun T. Jiang C. (2018). Biomacromolecules as Carriers in Drug Delivery and Tissue Engineering. Acta Pharmaceutica Sinica B 8 (1), 34–50. 10.1016/j.apsb.2017.11.005 29872621 PMC5985630

