# Supplementary material for: New Chitosan Polymer Scaffold Schiff Bases as Potential Cytotoxic Activity: Synthesis, Molecular Docking, and Physiochemical Characterization
Source: Front Chem. 2022 Jan 17;9:796599. doi: 10.3389/fchem.2021.796599 (PMC8801607; doi:10.3389/fchem.2021.796599)

Supporting Information

New Chitosan Polymer Scaffold Schiff Bases as Potential Cytotoxic activity: Synthesis, Molecular Docking, and Physiochemical characterization

**Ponnusamy Packialakshmi^1^, Perumal Gobinath^1^, Daoud Ali^2^, Saud Alarifi^2^, Raman Guruamy3, Akbar Idhayadhulla1, and RadhakrishnanSurendrakumar1***

Content Page. No.

^1^H-NMR Spectrum of compounds 2-11

^13^C-NMR Spectrum of the compounds 12-21

1H-NMR-Spectra

Compound-2a


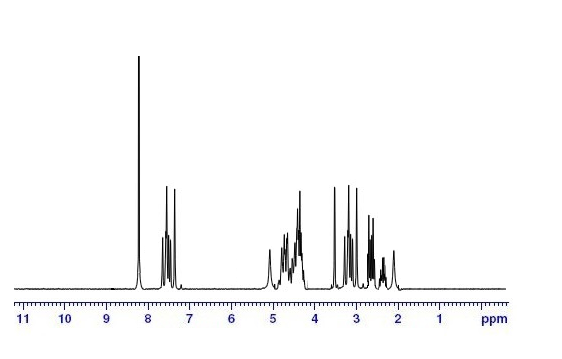


Compound-2b


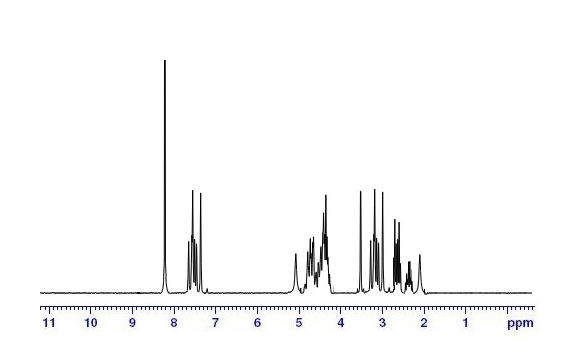


Compound-2c


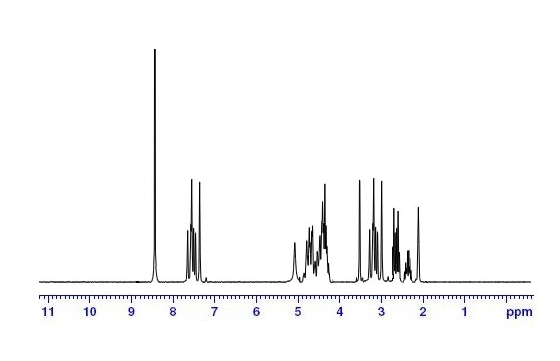


Compound-2d


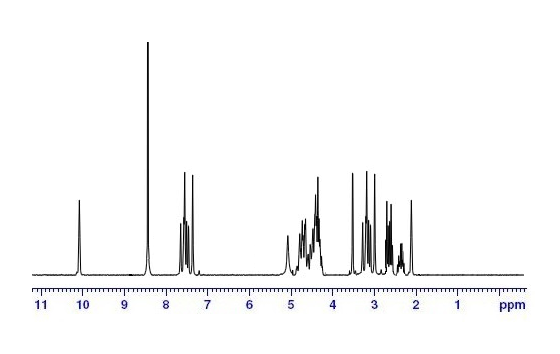


Compound-2e


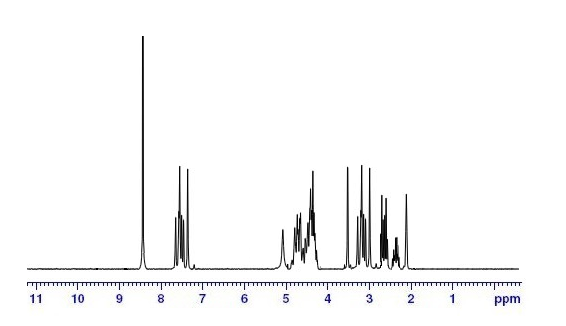


Compound-2f


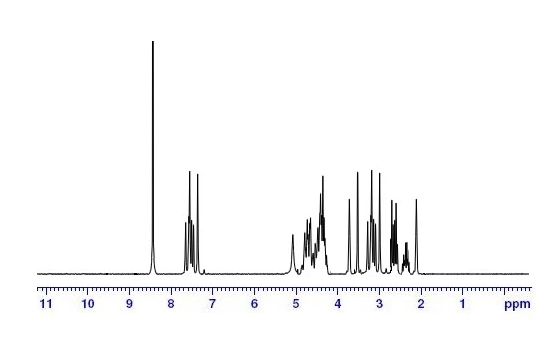


Compound-2g


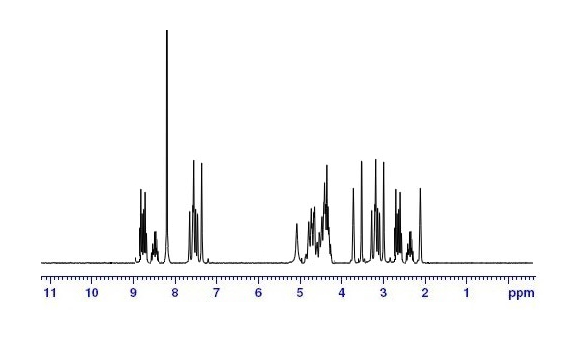


Compound-2h


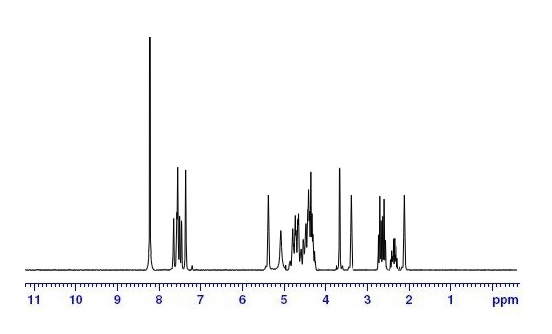


Compound-2i


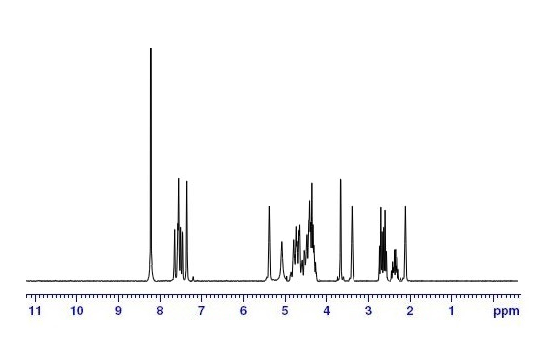


Compound-2j


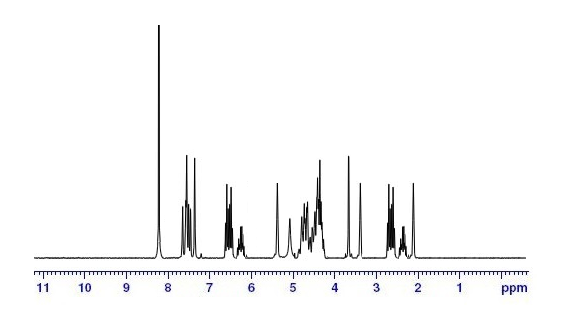


^13^C-NMR Spetcrum

Compound-2a


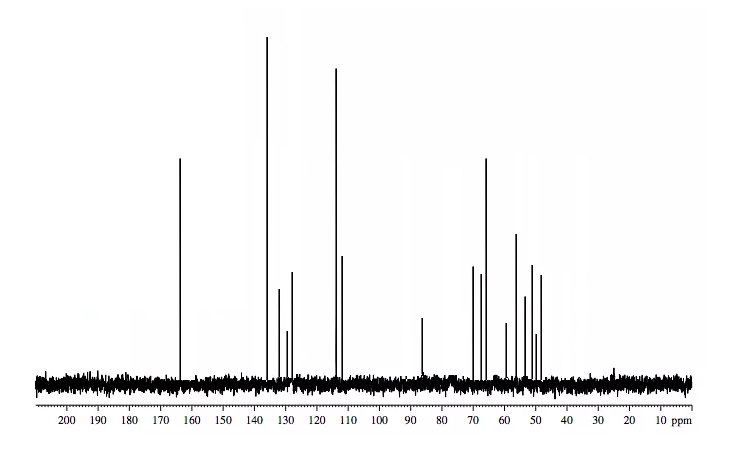


Compound 2b


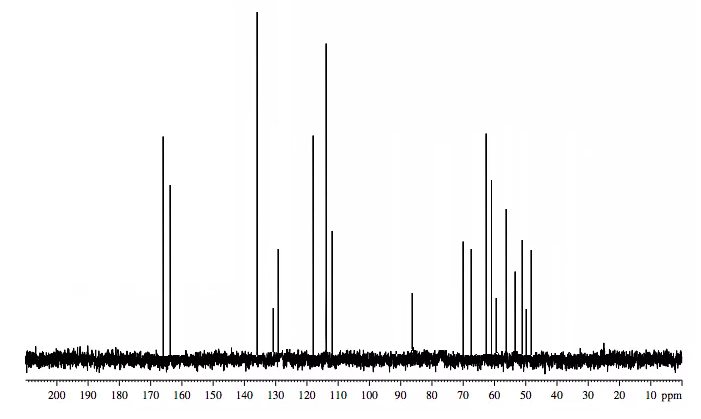


Compound -2c


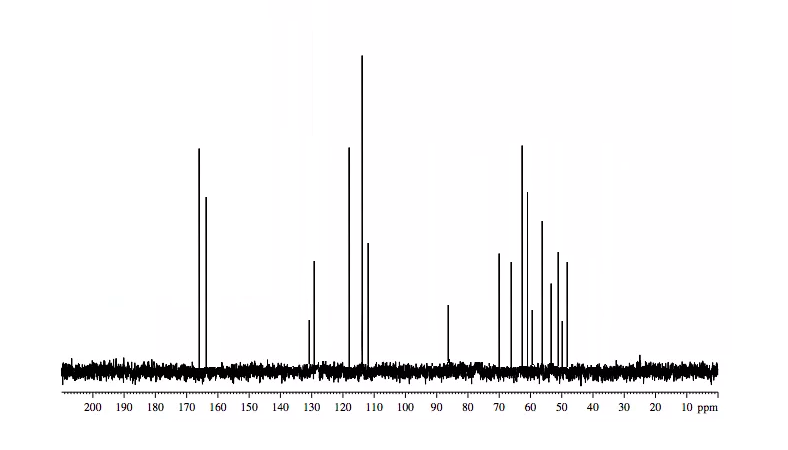


Compound-2d


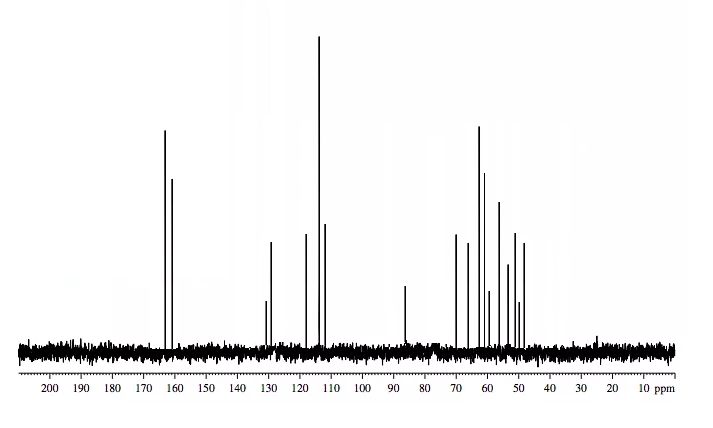


Compound-2e


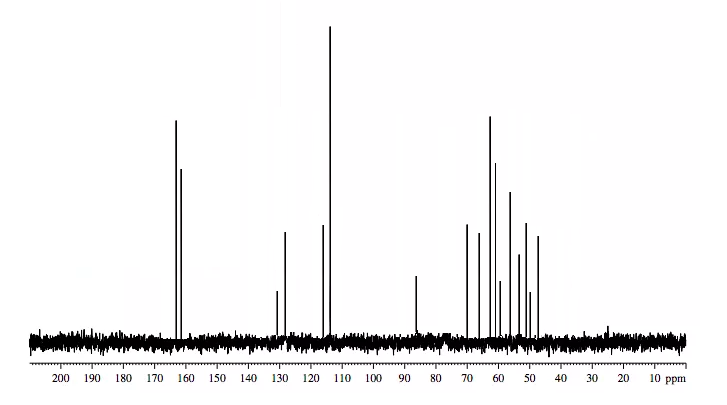


Compound -2f


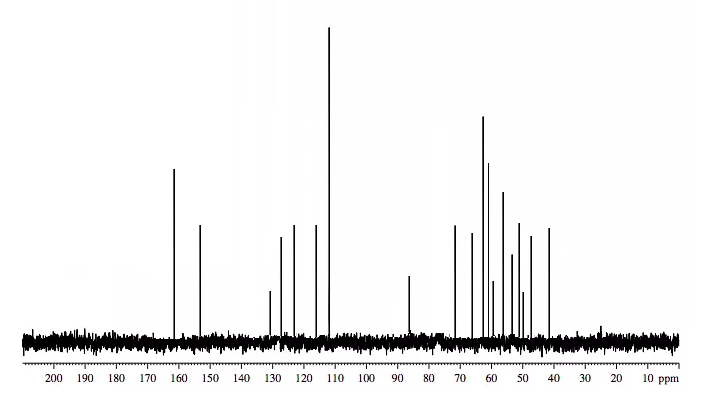


Compound-2g


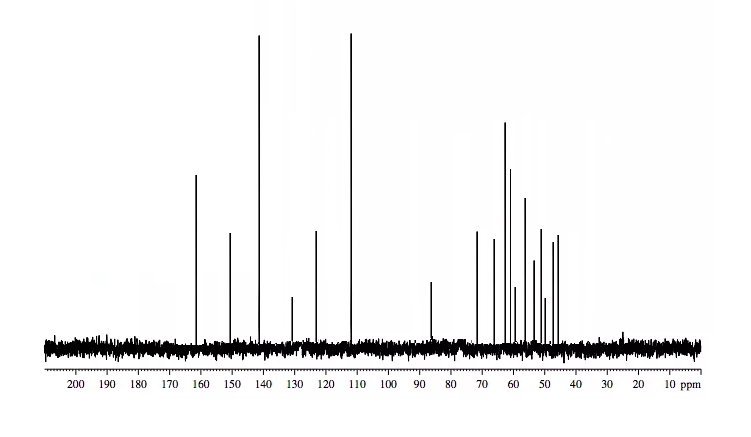


Compound -2h


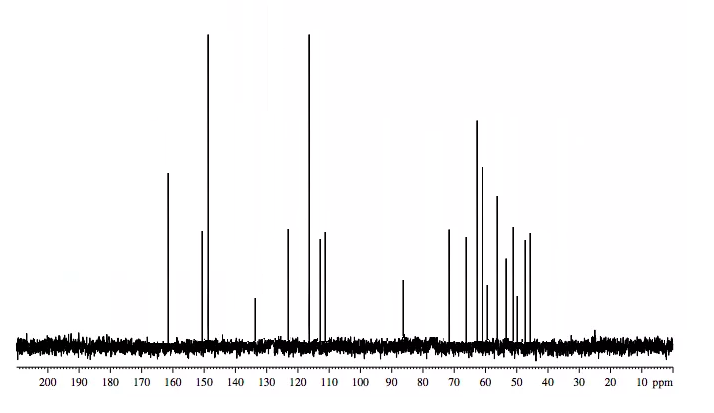


Compound -2i


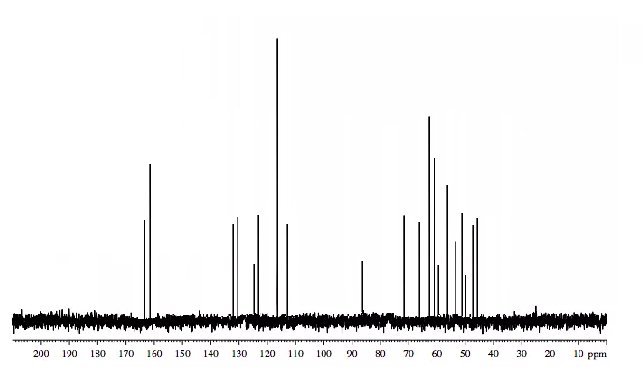


Compound-2j


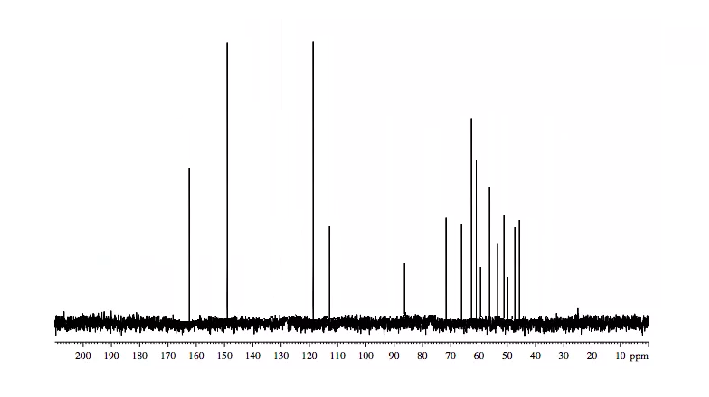

Supplement: Supplementary file 1 [file DataSheet1.doc]
